# Metformin Therapeutic Targets for Aortic Aneurysms: A Mendelian Randomization and Colocalization Study

**DOI:** 10.31083/j.rcm2503089

**Published:** 2024-03-05

**Authors:** Jingwen Liu, Mingyuan Xu, Bin Ni, Zhaohua Zhang, Xixi Gao, Dingkai Zhang, Liang Yang, Zhidong Ye, Jianyan Wen, Peng Liu

**Affiliations:** ^1^Peking University China‐Japan Friendship School of Clinical Medicine, 100029 Beijing, China; ^2^Department of Cardiovascular Surgery, China-Japan Friendship Hospital, 100029 Beijing, China; ^3^China-Japan Friendship Hospital (Institute of Clinical Medical Sciences), Chinese Academy of Medical Sciences & Peking Union Medical College, 100029 Beijing, China

**Keywords:** aortic aneurysm, colocalization, metformin, Mendelian randomization

## Abstract

**Background:**

Identifying effective pharmacological interventions to prevent the progressive enlargement and rupture of aortic aneurysms (AAs) is critical. Previous studies have suggested links between metformin use and a decreased incidence of AAs. In this study, we employed Mendelian randomization (MR) to investigate causal effects of metformin’s targets on AA risk and to explore the underlying mechanisms underlying these effects.

**Methods:**

To examine the relationship between metformin use and AA risk, we implemented both two-sample MR and multivariable MR analyses. Utilizing genetic instrumental variables, we retrieved cis-expression quantitative trait loci (cis-eQTL) data for potential targets of metformin from the Expression Quantitative Trait Loci Genetics Consortium (eQTLGen) Consortium and Genotype-Tissue Expression (GTEx) project. Colocalization analysis was employed to ascertain the probability of shared causal genetic variants between single nucleotide polymorphisms (SNPs) associated with eQTLs and AA.

**Results:**

Our findings reveal that metformin use reduces AA risk, exhibiting a protective effect with an odds ratio (OR) of 4.88 × ${\mathrm{10}}^{-3}$ (95% confidence interval [CI]: 7.30 × ${\mathrm{10}}^{-5}$–0.33, *p* = 0.01). Furthermore, the protective effect of type 2 diabetes on AA risk appears to be driven by metformin use (${\mathrm{OR}}_{\text{MVMR}}$ = 1.34 × ${\mathrm{10}}^{-4}$, 95% CI: 3.97 × ${\mathrm{10}}^{-8}$–0.45, *p* = 0.03). Significant Mendelian randomization (MR) results were observed for the expression of two metformin-related genes in the bloodstream: *NADH:ubiquinone oxidoreductase subunit A6* (*NDUFA6*) and *cytochrome b5 type B* (*CYB5B*), across two independent datasets (${\mathrm{OR}}_{\text{CYB5B}}$ = 1.35, 95% CI: 1.20–1.51, *p* = 2.41 × ${\mathrm{10}}^{-7}$; ${\mathrm{OR}}_{\text{NDUFA6}}$ = 1.12; 95% CI: 1.07–1.17, *p* = 1.69 × ${\mathrm{10}}^{-6}$). The MR analysis of tissue-specific expression also demonstrated a positive correlation between increased NDUFA6 expression and heightened AA risk. Lastly, NDUFA6 exhibited evidence of colocalization with AA.

**Conclusions:**

Our study suggests that metformin may play a significant role in lowering the risk of AA. This protective effect could potentially be linked to the mitigation of mitochondrial and immune dysfunction. Overall, *NDUFA6* has emerged as a potential mechanism through which metformin intervention may confer AA protection.

## 1. Introduction

An aortic aneurysm (AA) is characterized by localized dilations in the aorta, caused by thinning of the medial and adventitial layers due to extracellular matrix degradation and loss of vascular smooth muscle cells [1]. The rupture of large aneurysms can result in life-threatening hemorrhage and significantly increased mortality rates [2]. Established risk factors for AA encompass advanced age, male sex, hypertension, hyperlipidemia, and tobacco use [2]. At present, the standard for AA treatment is open or endovascular surgical intervention [3]. Consequently, a crucial clinical objective is to identify effective pharmaceutical treatments that can slow or prevent the growth and rupture of AAs [3].

Metformin, a widely used oral antihyperglycemic agent, is the primary pharmacological intervention for management of type 2 diabetes [4]. Its modulation of the inflammatory response has led to its consideration as a potential therapeutic target across a spectrum of cardiovascular conditions [5]. Epidemiological observations have indicated that metformin protects against the initiation and growth of AAs [6, 7], underscoring its possible utility in AA management. However, the challenge in drawing causal inferences from epidemiological studies lie in the presence of confounders, potential reverse causation, and confounding biases [5]. Furthermore, it is important to ascertain if the use of metformin can mitigate AA enlargement, in both diabetic and non-diabetic individuals. Consequently, a comprehensive investigation is warranted to determine if there is a causal link between metformin use and AA susceptibility.

Mendelian randomization (MR) is a method that uses single nucleotide polymorphisms (SNPs) as instrumental variables (IVs) to reduce the impact of confounding biases inherent to conventional observational studies [8]. Furthermore, integrating molecular phenotypes, including gene expression profiles with advanced techniques like genetic colocalization [9], have gained traction for identifying tissue-specific causal genes implicated in complex diseases. Such techniques are pivotal in unraveling the biological mechanisms of metformin’s impact on AA.

In this study, our objectives are to determine the causal impact of metformin on the risk of AA and to uncover the underlying therapeutic mechanisms behind these effects through MR and colocalization analyses. Our ultimate aim is to explore the potential of metformin as a potential therapeutic agent for AA.

## 2. Materials and Methods

### 2.1 Study Design

**Fig. 1.**
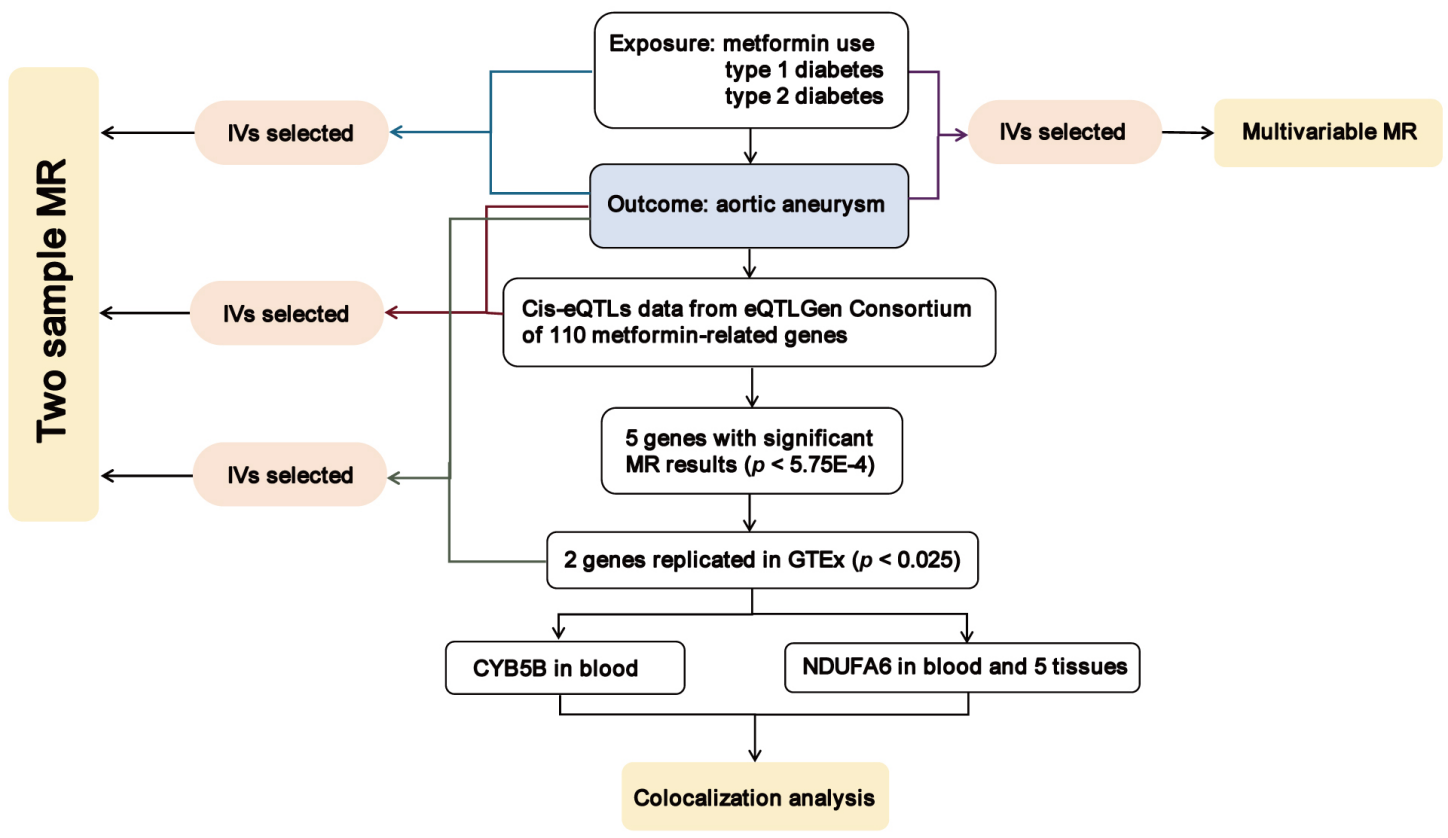
**Overview of the study design in our study**. MR, Mendelian randomization; IVs, instrumental variables; cis-eQTL, cis-expression quantitative trait loci; CYB5B, cytochrome b5 type B; NDUFA6, NADH:ubiquinone oxidoreductase subunit A6; GTEx, Genotype-Tissue Expression.

Our study’s primary objective was to assess the causal impact of metformin on AA risk and investigate the associated therapeutic targets relevant to metformin’s mechanism of action. The flow diagram illustrating the study methodology is presented in Fig. 1. Our approach involved the utilization of both two-sample MR and multivariable MR techniques to explore the causal relationships between metformin use, type 1 diabetes, type 2 diabetes, and AA risk. Furthermore, we employed a two-sample MR approach to assess the effects of gene-expression levels of metformin-related targets on AA risk, utilizing independent cis-expression quantitative trait (locicis-eQTL) variants. Subsequent steps were taken only for MR outcomes that achieved statistical significance, followed by validation in an independent dataset. Finally, upon identifying potential causal genetic variants, we performed a colocalization analysis to elucidate shared regulatory mechanisms underlying the observed effects.

### 2.2 Data Sources and Selection of Instrumental Variables

For the metformin exposure dataset, we utilized genome-wide association study (GWAS) summary statistics involving a total of 462,933 individuals, which comprises 11,552 cases and 451,381 controls. Participants were categorized based on their metformin treatment status, irrespective of age and sex. The type 1 diabetes and type 2 diabetes datasets encompassed 189,302 individuals (6729 cases and 182,573 controls) and 202,046 individuals (17,268 cases and 184,778 controls), respectively. The primary outcome was AA, which was defined based on diagnoses recorded in hospital episodes using the ICD-10 system, as well as the underlying and contributing causes of death documented with ICD-10 codes. The AA outcome dataset included GWAS summary statistics for 209,366 individuals (2825 cases and 206,541 controls). All datasets were exclusively of European origin and were obtained from the MRC-IEU OpenGWAS database, originating from the UK Biobank and FinnGen biobank. Refer to **Supplementary Table 1** for comprehensive details.

We retained SNPs associated with metformin use, type 1 diabetes, and type 2 diabetes at the genome-wide significance threshold (*p* < 5 × ${\mathrm{10}}^{-8}$), ensuring minimal linkage disequilibrium (LD) with other SNPs ($r^{2}$ < 0.001 within a clumping window of 10,000 kb). We also used the PhenoScanner software (http://www.phenoscanner.medschl.cam.ac.uk/) to examine confounder-associated phenotypes and outcomes [10, 11]. Employing the Mendelian Randomization Pleiotropy RESidual Sum and Outlier (MR-PRESSO) test, we detected potential horizontal pleiotropy which removed outliers and effectively controlled for pleiotropic effects [12].

In subsequent analyses, we obtained statistically significant cis-eQTL (false discovery rate <0.05, ±1 Mb from each probe) summary statistics from the Expression Quantitative Trait Loci Genetics Consortium (eQTLGen) consortium [13] and Genotype-Tissue Expression (GTEx) [14] version 8 for blood, and from GTEx for aorta, tibial artery, coronary artery, visceral omental adipose, and tibial nerve tissues. All eQTLs were cis-eQTLs, with SNPs extracted from 110 metformin-related genes based on a threshold of *p* < 5 × ${\mathrm{10}}^{-5}$ and $r^{2}$ < 0.1 within a clumping window of 100 kb, utilizing the 1000 Genomes European reference panel.

F-statistics were employed to gauge the strength of the selected IVs [15] and were calculated as follows: F = $R^{2}$(N-K-1)/[K(1-$R^{2}$)], where $R^{2}$ represents the proportion of exposure variance accounted for by the IVs, N signifies the effective sample size, and K denotes the number of variants incorporated in the IV model. An F-statistic exceeding 10 represented a robust correlation between the IVs and exposure [12]. SNPs with a minor allele frequency (MAF) of ≤0.01 were excluded.

### 2.3 Selection of Metformin Targets

We identified metformin targets through three distinct avenues: the drug-interaction database (DGldb) [16], the connectivity map (CMap) [17], and other pertinent literature [18, 19, 20, 21]. We encompassed all genes within DGldb that were implicated in potential metformin interactions. Genes with a connectivity score exceeding 90% or falling below –90% in response to metformin administration were selected. Additionally, we searched potential drug targets of metformin from other relevant literature. Genes meeting the aforementioned criteria were retained for subsequent analyses (**Supplementary Table 2)**.

### 2.4 Mendelian Randomization Analysis

Our two-sample MR approach was based on three fundamental assumptions: (1) Direct relevance of IVs to exposure; (2) Independence of IVs from confounding variables; (3) Influence of IVs on results through exposure [22].

The inverse-variance weighted (IVW) MR method was employed as the primary analysis, serving to quantify the strength of the association between exposure and outcome. Supplementary analyses encompassed MR-Egger, weighted median, simple mode, and weighted mode. Wald ratios were calculated for each SNP, providing individual estimates of the causal effect between exposure and outcome. For multivariable Mendelian randomization (MVMR), regression-based IVW and MR-Egger methodologies were utilized.

### 2.5 Sensitivity Analysis

To assess IV heterogeneity, Cochran’s Q test was used. Random-effects IVW was adopted when heterogeneity was detected. Conversely, fixed-effects IVW was employed when there was no heterogeneity. The intercept of the MR-Egger regression estimated the potential impact of pleiotropic effects from genetic variants on causal estimates. Furthermore, a leave-one-out sensitivity analysis was conducted to determine the susceptibility of MR results to individual SNPs. Verification of the directional causality from exposure to AA was facilitated through the MR Steiger test.

### 2.6 Colocalization Analysis

The colocalization analysis estimated the likelihood of shared causal variants between cis-eQTLs (as identified via MR) and AA risk. Genes meeting Bonferroni-corrected significance thresholds in the MR analysis for metformin-related genes underwent colocalization analysis for AA risk using default priors. The first prior, which was the likelihood of any variant serving as a causal determinant for any trait (H1 and H2), was set at 1 × ${\mathrm{10}}^{-4}$. The second prior, indicating the probability of a shared causal genetic instrument for both traits (H4), was established at 1 × ${\mathrm{10}}^{-5}$ [9]. Colocalization evidence was interpreted when the posterior probability for a shared causal variant exceeded 0.75 (posterior probability of hypothesis 4 >0.75). The analysis incorporated a range of distances from 70 kb to 2000 kb, given the limited number of IVs.

### 2.7 R Packages

All analytical procedures were executed using the R software (version 4.1.2, R Foundation for Statistical Computing, Vienna, Austria, https://www.r-project.org) and the following R packages: TwoSampleMR (version 0.5.7, https://mrcieu.github.io/TwoSampleMR/), MendelianRandomization (version 0.8.0, https://cran.r-project.org/web/packages/MendelianRandomization/) [23], and Coloc (version 5.2.2, https://chr1swallace.github.io/coloc/index.html) for colocalization analyses [9].

## 3. Results

### 3.1 Causal Effects of Metformin Treatment on AA Risk

**Fig. 2.**
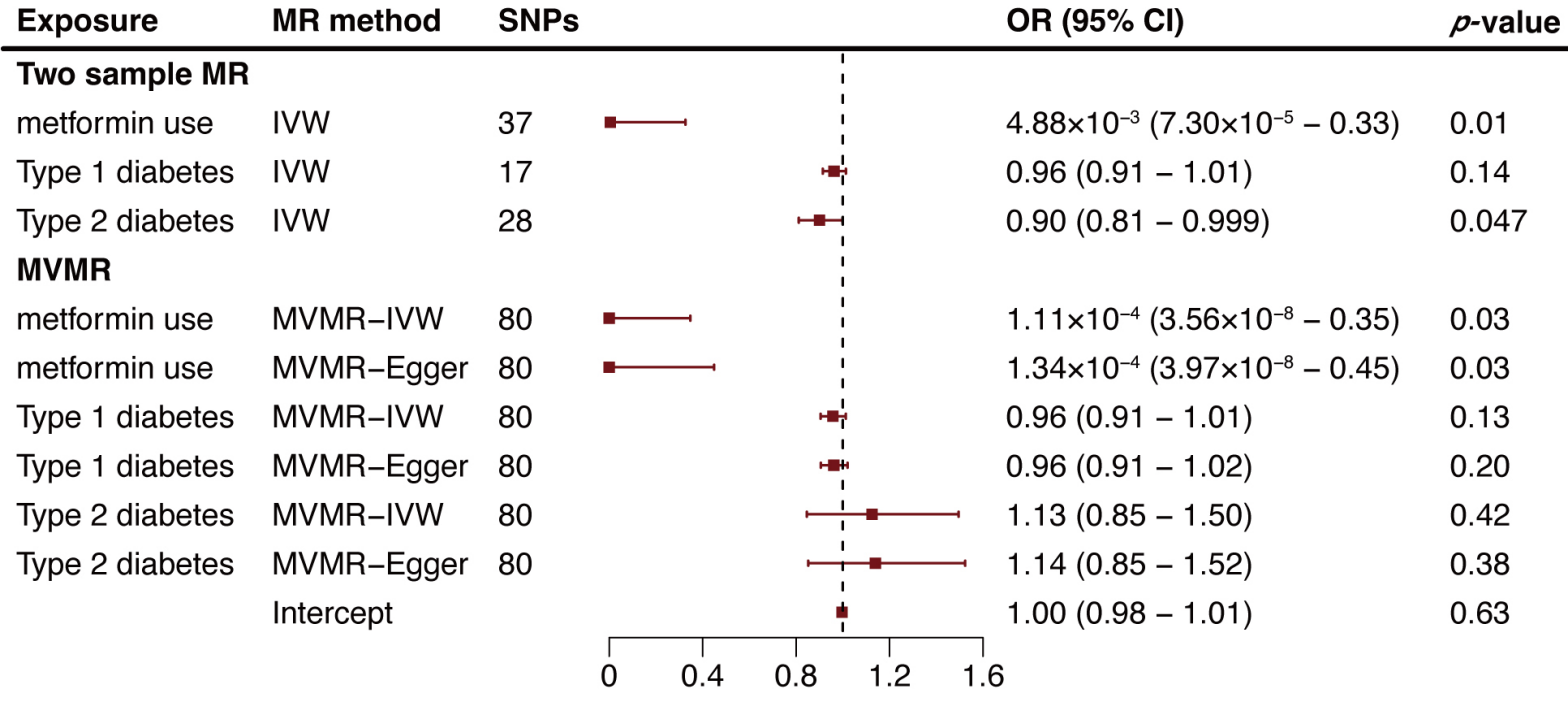
**Two-sample and multivariable mendelian randomization analysis results for metformin use, type 1 diabetes, and type 2 diabetes on aortic aneurysm risk**. MR, mendelian randomization; SNP, single nucleotide polymorphism; OR, odds ratio; CI, confidence intervals; IVW, inverse variance weighted method; MVMR, multivariable Mendelian randomization.

The two-sample MR analysis suggested a potential negative association between metformin use and AA risk, with metformin users showing a reduced risk (odds ratio [OR] = 4.88 × ${\mathrm{10}}^{-3}$, 95% confidence interval [CI]: 7.30 × ${\mathrm{10}}^{-5}$–0.33, *p *= 0.01; Fig. 2; **Supplementary Fig. 1**). A similar protective trend was observed in type 2 diabetes patients (OR = 0.90, 95% CI: 0.81–0.999, *p* = 0.047; Fig. 2; **Supplementary Table 3**). Conversely, no significant relationship was established between type 1 diabetes and AA (*p* = 0.14; Fig. 2; **Supplementary Table 3**). Further details on all SNPs are provided in** Supplementary Table 4**. Evaluation of SNPs for heterogeneity through Cochran’s Q test yielded no significant findings (**Supplementary Table 5**). No outliers were identified via MR-PRESSO, and MR-Egger regression analysis detected no directional pleiotropy (**Supplementary Table 5**). The MR Steiger test confirmed that exposure causally influenced AAs (**Supplementary Table 5**). It is important to note that interpreting metformin’s effect on non-diabetic individuals remains challenging due to pleiotropic effects. To address this, multivariable MR (MVMR) was employed, a recent MR extension that accounts for pleiotropy among multiple traits [24]. In this context, MVMR analysis indicated that the protective effect initially associated with type 2 diabetes was no longer present (OR = 1.14, 95% CI: 0.85–1.52, *p* = 0.38; Fig. 2, **Supplementary Table 6**). In contrast, the protective effect linked to metformin persisted (OR = 1.34 × ${\mathrm{10}}^{-4}$, 95% CI: 3.97 × ${\mathrm{10}}^{-8}$–0.45, *p* = 0.03; Fig. 2, **Supplementary Table 6**). This suggests that the observed protective effect of type 2 diabetes on AA may be a result of metformin treatment.

### 3.2 Effect of Metformin Therapeutic Target Gene Expression Levels in the Whole Blood on AA Risk

To identify genes potentially affected by metformin treatment that may reduce AA risk, we compiled data on genes interacting with metformin, gathering 38 from DGldb, 20 from CMap, and 52 from relevant literature (**Supplementary Table 2**). We then selected conditionally independent cis-expression quantitative trait loci (cis-eQTL) variants that correlated with whole blood concentrations from the eQTLGen. Following this selection, we focused on 87 metformin-related genes, examining their mRNA expression in relation to AA risk. We employed a two-sample MR approach based on European summary statistics for AA patients. After multiple testing adjustments (*p* < 5.75 × ${\mathrm{10}}^{-4}$ [IVW], Bonferroni-corrected to 0.05 for 87 genes), we identified 5 cis-eQTLs associated with AAs risk. Notably, increased *cytochrome b5 type B* (*CYB5B*) and *NADHL: ubiquinone oxidoreductase subunit A6* (*NDUFA6*) levels in blood were linked to heightened risk (${\mathrm{OR}}_{\text{CYB5B}}$ = 1.35, 95% CI: 1.20–1.51, *p* = 2.40 × ${\mathrm{10}}^{-7}$; ${\mathrm{OR}}_{\text{NDUFA6}}$ = 1.11, 95% CI: 1.07–1.17, *p* = 1.69 × ${\mathrm{10}}^{-6}$; Table 1; **Supplementary Table 7**). Conversely, changes in *protein kinase AMP-activated catalytic subunit alpha 1* (*PRKAA1*), *high mobility group box 3* (*HMGB3*), and *NADH:ubiquinone oxidoreductase complex assembly factor 3* (*NDUFAF3*) levels in blood were associated with reduced AA risk (Table 1; **Supplementary Table 7**). Our results for *CYB5B* and *NDUFA6* were further validated using cis-eQTL data from the Genotype-Tissue Expression (GTEx) Project (no valid SNPs were available for analysis of the other three genes). Notably, these two targets retained statistical significance even after applying a stringent Bonferroni threshold (${\mathrm{OR}}_{\text{CYB5B}}$ = 1.77, 95% CI: 1.14–2.74, *p* = 0.01; ${\mathrm{OR}}_{\text{NDUFA6}}$ = 1.29, 95% CI: 1.16–1.45, *p* = 6.76 × ${\mathrm{10}}^{-6}$; *p* < 0.025 [IVW/Wald ratio], Bonferroni correction for 0.05/2 genes; Table 1; **Supplementary Table 8**). Importantly, the direction of effect exhibited 100% consistency (**Supplementary Tables 9, 10**), and no signs of excess heterogeneity or pleiotropy were evident (**Supplementary Tables 9, 10**).

**Table 1.** **Mendelian randomization results of genes in whole blood**.

Genes	eQTLGen Consortium					GTEx Project				
	SNPs	OR (95% CI)	IVW/Ward Ratio	MR-Egger intercept	Egger intercept	SNPs	OR (95% CI)	IVW/Ward Ratio	MR-Egger intercept	Egger intercept
			*p*-value		*p*-value			*p*-value		*p*-value
P*RKAA1*	12	0.65 (0.52–0.81)	1.08 × ${\mathrm{10}}^{–\,4}$	–0.05	0.37					
*HMGB3*	1	0.09 (0.003–0.25)	6.29 × ${\mathrm{10}}^{–\,6}$	-	-					
*CYB5B*	39	1.35 (1.20–1.51)	2.41 × ${\mathrm{10}}^{–\,7}$	0.01	0.42	1	1.77 (1.14–2.74)	1.10 × ${\mathrm{10}}^{–\,2}$	-	-
*NDUFA6*	78	1.12 (1.07–1.17)	1.69 × ${\mathrm{10}}^{–\,6}$	–0.004	0.62	12	1.30 (1.16–1.45)	6.76 × ${\mathrm{10}}^{–\,6}$	0.02	0.44
*NDUFAF3*	47	0.70 (0.63–0.78)	1.89 × ${\mathrm{10}}^{–\,11}$	0.03	0.05					

eQTLGen, Expression Quantitative Trait Loci Genetics Consortium; GTEx, Genotype-Tissue Expression Project; SNP, single nucleotide polymorphism; OR, odds ratio; CI, confidence intervals; IVW, inverse variance weighted method; MR-Egger, Mendelian Randomization Egger; *PRKAA1*, *protein kinase AMP-activated catalytic subunit alpha 1*; *HMGB3*, *high mobility group box 3*; *CYB5B*, *cytochrome b5 type B*; *NDUFA6*, *NADH, ubiquinone oxidoreductase subunit A6*; *NDUFAF3*, *NADH:ubiquinone oxidoreductase complex assembly factor 3*.

### 3.3 Effect of NDUFA6 Expression Levels in 5 Tissues on AA Risk

**Fig. 3.**
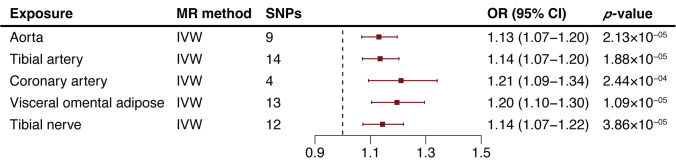
**Two-sample mendelian randomization analysis results of NADH:ubiquinone oxidoreductase subunit A6 (NDUFA6) in tissues on aortic aneurysm (AA) risk**. MR, Mendelian randomization; SNP, single nucleotide polymorphism; OR, odds ratio; CI, confidence intervals; IVW, inverse variance weighted method; AA, aortic aneurysm.

We extended our analysis by extracting *NDUFA6*-related cis-eQTLs from the GTEx Project, examining their impact across various tissues including the aorta, tibial artery, coronary artery, visceral omental adipose, and tibial nerve. The MR results revealed a consistent positive association between increased *NDUFA6* expression in arterial tissues and elevated AA risk. Specifically, the ORs were 1.13 for aorta (95% CI: 1.07–1.20, *p* = 2.13 × ${\mathrm{10}}^{-5}$), 1.14 for tibial artery (95% CI: 1.07–1.20, *p* = 1.88 × ${\mathrm{10}}^{-5}$), and 1.21 for coronary artery (95% CI: 1.09–1.34, *p* = 2.44 × ${\mathrm{10}}^{-4}$; Fig. 3; **Supplementary Table 11**). Furthermore, elevated *NDUFA6* expression in visceral omental adipose and nerve tissues also correlated with increased AA risk (${\mathrm{OR}}_{\text{visceral omental adipose}}$ = 1.20, 95% CI: 1.19–1.30, *p* = 2.09 × ${\mathrm{10}}^{-5}$; ${\mathrm{OR}}_{\text{tibial nerve}}$ = 1.14, 95% CI: 1.07–1.22, *p* = 3.86 × ${\mathrm{10}}^{-5}$; Fig. 3; **Supplementary Table 11**). Sensitivity analysis findings are detailed in **Supplementary Table 12**. No evidence of heterogeneity or pleiotropy was observed in the MR analyses of *NDUFA6* expression levels on AA risk (**Supplementary Table 12**). The Steiger directionality test further supported that the genetic associations aligned with a causal impact of *NDUFA6* levels on AA risk, rather than the inverse (**Supplementary Table 12**). Refer to **Supplementary Table 13 **for a comprehensive list of IVs. Notably, no cis-eQTLs related to *CYB5B* were identified across the tissues analyzed.

### 3.4 Colocalization Analysis

Our colocalization analysis probed the likelihood of shared causal genetic variants between SNPs associated with AAs and eQTLs. Intriguingly, our findings indicated a shared causal variant between AAs and eQTLs for both *CYB5B* and *NDUFA6* in blood with posterior probabilities of 0.77 and 0.99 respectively (Table 2; **Supplementary Table 14**). Notably, two cis-eQTLs of *NDUFA6* in the tibial artery and visceral omental adipose also presented compelling evidence for a shared causal variant with AA, each with a posterior probability 1 (Table 2; **Supplementary Table 14**). These results, derived from both MR and colocalization analyses highlighted two potentially metformin-related genes with a possible shared genetic influence between eQTLs and AAs risk.

**Table 2.** **Colocalization analysis: gene expression and AA risk with a high posterior probability of shared causal variants >0.75 between expression and AA risk**.

Gene	Tissue	Variants	SNP	Position	Alleles	EAF	Beta	PPH4
*CYB5B*	Blood	534	rs72797202	16:69519790	A/C	0.21	0.14; 0.05	0.77
*NDUFA6*	Blood	1340	rs117529804	22:42669104	T/C	0.04	1.03; 0.18	0.99
*NDUFA6*	Tibial artery	431	rs1989202	22:42486080	A/G	0.02	1.41; 0.15	1.00
*NDUFA6*	Visceral omental adipose	304	rs1989202	22:42486080	A/G	0.02	0.95; 0.15	1.00

SNP means the shared causal variant (PPH >0.75); Position, chromosome and base pair position; Alleles indicates effect allele/other allele; EAF, effect allele frequency; Beta, effect size estimates of exposure and outcome, respectively; PPH4, posterior probability of a shared causal variant (hypothesis 4); SNP, single nucleotide polymorphism; *CYB5B*, *cytochrome b5 type B*; *NDUFA6*, *NADH:ubiquinone oxidoreductase subunit A6*; AA, aortic aneurysm.

## 4. Discussion

This study investigated the causal effects of metformin use on AA risk as well as the therapeutic mechanisms by employing two-sample and multivariable MR analyses. We established that the observed protective effect of type 2 diabetes on AA is likely due to metformin use. By using both eQTL and colocalization analyses, we discovered that variations in *CYB5B* and *NDUFA6* gene expression, present in blood and tissue, may provide insight into metformin’s protective effect on AA.

The paradoxical protective effect of type 2 diabetes on AAs has been substantiated by numerous studies, in both observational studies and MR investigations [25, 26]. This phenomenon challenges the conventional understanding that elevated glucose levels promote endothelial and platelet dysfunction [27], leading to chronic inflammation in vessel walls [28]—a pivotal factor in AA development [29, 30]. Even with careful glycemic control, other factors such as hypertension, can lead to vascular inflammation and AA development [31]. Various antidiabetic treatments, including metformin, have been linked to reduced AA risk [7]. Among these, metformin represents a long-standing, well-established pharmacological intervention with proven safety and efficacy. While observational studies have explored its effects on AA, our study used MR to avoid potential confounders, reverse causation, and biases inherent in traditional observational research, thus providing a more robust assessment of metformin’s impact on AA.

The protective effects of both type 2 diabetes and metformin on AA, initially supported by two-sample MR, exhibited notable differences when analyzed using MVMR. In MVMR, the protective effect attributed to type 2 diabetes diminished, while the influence of metformin remained consistent. This observation suggests that the apparent protective effect of type 2 diabetes on AA might indeed stem from metformin treatment.

The effort to reveal the underlying mechanisms by which metformin may mitigate AA risk holds significant importance. Apart from its effects on glucose metabolism, metformin’s influence on immunometabolism has positioned it as a promising target for treating cardiovascular disease [32]. Experimental animal models have illustrated metformin’s role in preserving medial elastin and smooth muscle while reducing immune cell infiltration, ultimately inhibiting aortic aneurysm formation and progression [33]. Moreover, patient-derived microphysiological models underscore metformin’s potential to regulate contractile phenotypes and metabolic abnormalities in aortic smooth muscle cells, effectively reducing aortic dilation [34].

We explored three pathways to investigate metformin targets: DGldb, CMap, and other pertinent literature [20]. Notably, a significant portion of these targets were related to *mitochondrial complex I* (*MCI*) and *adenosine monophosphate-activated protein kinase* (*AMPK*) families, which intrinsically correlate with their anti-inflammatory mechanisms [35]. Through the integration of MR with GWAS datasets and eQTL, our analysis yielded significant outcomes for *NDUFA6* and *CYB5B*.

*NDUFA6* encodes a pivotal accessory subunit of MCI (NADH:ubiquinone oxidoreductase), the principal enzyme within the mitochondrial membrane respiratory chain responsible for energy conversion [36, 37]. Notably, MCI also emerges as a primary source of reactive oxygen species (ROS), which have been implicated in the regulation of the NLRP3 inflammasome [38]. In line with our study, higher levels of NDUFA6 in blood were associated with an elevated risk of AA. This trend persisted across various tissues, including aorta, tibial artery, coronary artery, visceral omental adipose, and tibial nerve. These tissues are all potential sites of metformin action [39, 40]. Existing literature hints at metformin’s role in inhibiting MCI activity and cellular respiration [41, 42]. Intriguingly, our colocalization analysis pinpointed shared causal variants in blood, tibial artery, and visceral omental adipose that further suggested an intricate interplay between NDUFA6 expression across diverse tissues and AA risk. This finding provides support for the idea that metformin’s possible inhibition of MCI activity, and even mitochondrial ATP synthase [43], could be mediated through NDUFA6 suppression.

In addition to investigating *NDUFA6* expression in various arterial tissues (aorta, tibial artery, coronary artery), our study also explored the visceral omental adipose and tibial nerve (limited to available data). The consistent elevation in *NDUFA6* levels across these tissues correlated with increased AA risk. The link between perivascular adipose tissue (PVAT) and AA pathophysiology has been provided, given PVAT’s status as a paracrine organ that produces inflammatory agents with potential vascular impact [44]. Similarly, studies have hinted at the association between PVAT deposition and abdominal aortic aneurysm (AAA) pathology [45, 46], with metformin reducing visceral fat and adipose inflammation [47, 48]. Increased overall and regional aortic sympathetic nervous system activities within aortic lesions has been observed, along with metformin’s impact on peripheral nerve recovery [49, 50]. While not directly parallel to interactions in PVAT and the sympathetic nervous system, our findings offer mechanistic insights into metformin’s potential risk reduction in AA.

CYB5B, primarily located on the outer mitochondrial membrane, plays a role in the heme-dependent nitric oxide biosynthetic process [51, 52]. A study demonstrated the potential to enrich mitochondria using anti-CYB5B immunomagnetic beads, offering a viable method for mitochondrial micro-preparation from cultured cells [53]. Additionally, CYB5B overexpression has been identified in Hodgkin Lymphoma and aggressive Non-Hodgkin Lymphomas [54], further underscoring its link to mitochondria and immune response. Single-cell RNA sequencing studies have also revealed heightened immune cell presence and extensive mitochondrial dysfunction within ascending thoracic aortic aneurysm tissues [55]. Our study’s observation of elevated CYB5B levels in blood correlating with increased AA risk aligns with this framework. However, the absence of eQTL data for CYB5B across various tissues hinders our understanding of this gene’s specific role.

This study has several strengths. It elucidates the association between metformin and AA using the potent MR method, which effectively overcomes the potential influence of confounders, reverse causation, and traditional observational biases. Furthermore, we reveal the protective effect of type 2 diabetes on AA, attributing it to metformin and substantiating its mediating role. The identification of other possible metformin targets and their variants provides a robust foundation for guiding future drug repurposing studies.

Nevertheless, certain limitations should be acknowledged. The persistent influence of directional pleiotropy cannot be entirely ruled out due to the varying dosages of metformin across studies. Moreover, since the data we used lacked information on metformin dosage, patient sex, and lesion location, we were unable to conduct subgroup analysis to assess whether the protective effect of metformin on AA differed based on lesion site, sex, or dose-dependency. Another concern is that MR gene expression analysis depends on a limited set of genetic predictors, which raises potential issues about the bias of weak instrumental variables. In addition, the complex mechanisms underlying metformin’s effects still remain unclear, which could result in the omission of relevant targets and genes. Further experimental investigations are warranted. Moreover, it’s important to note that the study was confined to individuals of European ancestry, which may limit the generalizability of findings to other ethnicities.

## 5. Conclusions

This comprehensive study establishes a foundation for exploring the effects of metformin, grounded in genetics. It provides evidence supporting metformin’s causal impact on diminishing AA risk within the European population. The underlying mechanisms appear to be linked to disruptions in mitochondrial and immune regulation, with the MCI-associated gene NDUFA6 emerging as a critical mediator. These findings not only underscore the potential of repurposing metformin but also highlight to researchers the significance of focusing on NDUFA6 as a metformin-associated target for the prevention of AA in the broader population.

## Data Availability

Data on exposures and outcome were contributed by a number of studies in the IEU OpenGWAS database and were downloaded from https://gwas.mrcieu.ac.uk/. eQTL data from version 8 EUR of the GTEx Project were downloaded in https://gtexportal.org/home/datasets. eQTL data from eQTLGen Consortium were downloaded in https://www.eqtlgen.org/cis-eqtls.html. We also used the Phenoscanner web-based resource (http://www.phenoscanner.medschl.cam.ac.uk/). This study was presented in accordance with the Strengthening the Reporting of Observational Studies in Epidemiology using Mendelian Randomization (STROBE-MR) which downloaded from https://www.strobe-mr.org (checklist shown in **Supplementary Table 15**). The data generated from this paper are available in the Tables and the Supplementary Information. The data underlying this article will be shared upon reasonable request to the corresponding author.

## Abbreviations

AA, aortic aneurysm; CI, confidence intervals; cis-eQTL, cis-expression quantitative trait loci; CYB5B, cytochrome b5 type B; EAF, effect allele frequency; GWAS, genome-wide association study; HMGB3, high mobility group box 3; IVW, inverse variance weighted method; LD, linkage disequilibrium; MR, Mendelian randomization; MR-PRESSO, Mendelian Randomization Pleiotropy RESidual Sum and Outlier; MVMR, multivariable Mendelian randomization; NDUFA6, NADH:ubiquinone oxidoreductase subunit A6; NDUFAF3, NADH:ubiquinone oxidoreductase complex assembly factor 3; OR, odds ratio; PRKAA1, protein kinase AMP-activated catalytic subunit alpha 1; SNP, single nucleotide polymorphisms.

## Supplementary Material

Supplementary material associated with this article can be found, in the online version, at https://doi.org/10.31083/j.rcm2503089.
