# Supplementary material for: Metformin Therapeutic Targets for Aortic Aneurysms: A Mendelian Randomization and Colocalization Study
Source: Rev Cardiovasc Med. 2024 Mar 5;25(3):89. doi: 10.31083/j.rcm2503089 (PMC11263823; doi:10.31083/j.rcm2503089)
Supplement: Supplementary file 1 [file 2153-8174-25-3-089-s1.docx]

**Metformin** **Therapeutic Targets** **for Aortic Aneurysms:** **a Mendelian Randomization and Colocalization Study**

**Supplementary Table 1. Data sources of metformin use, type 1 diabetes type 2 diabetes and aortic aneurysms in our study.**

**Supplementary Table 2. Metformin-related genes identified by DGIdb, Cmap and literatures.**

**Supplementary Table 3. Two-sample Mendelian randomization analysis of causal effects of metformin use, type 1 diabetes and type 2 diabetes on AA.**

**Supplementary Table 4. Independent instruments for two-sample MR analysis: harmonized data.**

**Supplementary Table 5. Sensitivity analysis of two-sample MR analysis.**

**Supplementary Table 6. Causal relationships of metformin use on AA adjusted for type 1 and 2 diabetes estimated by multivariable MR.**

**Supplementary Table 7. The results of 5 metformin target genes in blood and aortic aneurysm of Mendelian randomization in eQTLGen.**

**Supplementary Table 8. The results of 2 metformin target genes in blood and aortic aneurysm of Mendelian randomization in GTEx.**

**Supplementary Table 9. The results of heterogeneity, pleiotropy analysis and steiger test in eQTLGen.**

**Supplementary Table 10. The results of heterogeneity, pleiotropy analysis and steiger test in GTEx.**

**Supplementary Table 11. The results of NDUFA6 and aortic aneurysm of Mendelian randomization in 5 tissues in GTEx.**

**Supplementary Table 12. The results of heterogeneity, pleiotropy analysis and steiger test in 5 tissues.**

**Supplementary Table 13. A detailed summary of genetic variants in eQTL-MR analysis: harmonized data.**

**Supplementary Table 14. The results of colocalization analysis.**

**Supplementary Table 15. STROBE-MR checklist of recommended items to address in reports of Mendelian randomization studies**

**Supplementary Fig. 1. MR effect size for causal associations of metformin use and aortic aneurysm.**

**Supplementary Table 1. Data sources of metformin use, type 1 diabetes type 2 diabetes and aortic aneurysms in our study.**

| **Trait** | **Year** | **Phenotypic code** | **Sample Size** | **Case/controls** | **SNPs** | **Ancestry** |
| --- | --- | --- | --- | --- | --- | --- |
| Metformin use | 2018 | ukb-b-14609 | 462933 | 11552/451381 | 9851867 | European |
| Aortic aneurysms | 2021 | finn-b-I9_AORTANEUR | 209366 | 2825/206541 | 16380417 | European |
| Type 1 diabetes | 2021 | finn-b-T1D_WIDE | 189302 | 6729/182573 | 16380061 | European |
| Type 2 diabetes | 2021 | finn-b-T2D_WIDE | 202046 | 17268/184778 | 16380418 | European |

**Supplementary Table 2**. **Metformin-related genes identified by DGIdb, Cmap and literatures.**

| **Database** | **Gene name** | **ENSG.ID** |
| --- | --- | --- |
| DGldb | CAPN10 | ENSG00000142330 |
| DGldb | ATM | ENSG00000149311 |
| DGldb | AMHR2 | ENSG00000135409 |
| DGldb | C11ORF65 | ENSG00000166323 |
| DGldb | SLC22A1 | ENSG00000175003 |
| DGldb | PTEN | ENSG00000171862 |
| DGldb | MTOR | ENSG00000198793 |
| DGldb | PRKAA2 | ENSG00000162409 |
| DGldb | PRKAB1 | ENSG00000111725 |
| DGldb | PRKAG2 | ENSG00000106617 |
| DGldb | IDH1 | ENSG00000138413 |
| DGldb | SLC29A4 | ENSG00000164638 |
| DGldb | STK11 | ENSG00000118046 |
| DGldb | ACACB | ENSG00000076555 |
| DGldb | SLC22A2 | ENSG00000112499 |
| DGldb | SLC47A1 | ENSG00000142494 |
| DGldb | PRKAG1 | ENSG00000181929 |
| DGldb | SLC47A2 | ENSG00000180638 |
| DGldb | FMO5 | ENSG00000131781 |
| DGldb | PRKAA1 | ENSG00000132356 |
| DGldb | KRAS | ENSG00000133703 |
| DGldb | PRKAG3 | ENSG00000115592 |
| DGldb | SLC19A3 | ENSG00000135917 |
| DGldb | NRAS | ENSG00000213281 |
| DGldb | PPARA | ENSG00000186951 |
| DGldb | CPA6 | ENSG00000165078 |
| DGldb | PRKAB2 | ENSG00000131791 |
| DGldb | SLC22A3 | ENSG00000146477 |
| DGldb | HNF1B | ENSG00000276194 |
| DGldb | SLC2A2 | ENSG00000163581 |
| DGldb | NBEA | ENSG00000172915 |
| DGldb | KCNJ11 | ENSG00000187486 |
| DGldb | PRPF31 | ENSG00000105618 |
| DGldb | PIK3CA | ENSG00000121879 |
| DGldb | SP1 | ENSG00000185591 |
| DGldb | NR1I2 | ENSG00000144852 |
| DGldb | ERBB2 | ENSG00000141736 |
| DGldb | HRAS | ENSG00000174775 |
| CMap | UTP14A | ENSG00000156697 |
| CMap | CNOT8 | ENSG00000155508 |
| CMap | ARHGAP35 | ENSG00000160007 |
| CMap | HEXB | ENSG00000049860 |
| CMap | HMGB3 | ENSG00000029993 |
| CMap | CYB5B | ENSG00000103018 |
| CMap | RBCK1 | ENSG00000125826 |
| CMap | KRT25 | ENSG00000204897 |
| CMap | SMAD4 | ENSG00000141646 |
| CMap | NDUFB3 | ENSG00000119013 |
| CMap | SLC16A6 | ENSG00000108932 |
| CMap | PROKR1 | ENSG00000169618 |
| CMap | BCL6B | ENSG00000161940 |
| CMap | MMP1 | ENSG00000196611 |
| CMap | CTSK | ENSG00000143387 |
| CMap | MAGED1 | ENSG00000179222 |
| CMap | MAP3K5 | ENSG00000197442 |
| CMap | ATP5C1 | ENSG00000165629 |
| CMap | NKIRAS1 | ENSG00000197885 |
| CMap | INS | ENSG00000254647 |
| Literature | GPD2 | ENSG00000115159 |
| Literature | GDF15 | ENSG00000130513 |
| Literature | GCG | ENSG00000115263 |
| Literature | NDUFA10 | ENSG00000130414 |
| Literature | NDUFA11 | ENSG00000174886 |
| Literature | NDUFA12 | ENSG00000184752 |
| Literature | NDUFA13 | ENSG00000186010 |
| Literature | NDUFA2 | ENSG00000131495 |
| Literature | NDUFA3 | ENSG00000170906 |
| Literature | NDUFA4 | ENSG00000189043 |
| Literature | NDUFA4L2 | ENSG00000185633 |
| Literature | NDUFA5 | ENSG00000128609 |
| Literature | NDUFA6 | ENSG00000184983 |
| Literature | NDUFA7 | ENSG00000267855 |
| Literature | NDUFA8 | ENSG00000119421 |
| Literature | NDUFA9 | ENSG00000139180 |
| Literature | NDUFAB1 | ENSG00000004779 |
| Literature | NDUFAF1 | ENSG00000137806 |
| Literature | NDUFAF2 | ENSG00000164182 |
| Literature | NDUFAF3 | ENSG00000178057 |
| Literature | NDUFAF4 | ENSG00000123545 |
| Literature | NDUFB1 | ENSG00000183648 |
| Literature | NDUFB10 | ENSG00000140990 |
| Literature | NDUFB2 | ENSG00000090266 |
| Literature | NDUFB5 | ENSG00000136521 |
| Literature | NDUFB6 | ENSG00000165264 |
| Literature | NDUFB7 | ENSG00000099795 |
| Literature | NDUFB8 | ENSG00000166136 |
| Literature | NDUFB9 | ENSG00000147684 |
| Literature | NDUFC1 | ENSG00000109390 |
| Literature | NDUFC2 | ENSG00000151366 |
| Literature | NDUFS1 | ENSG00000023228 |
| Literature | NDUFS2 | ENSG00000158864 |
| Literature | NDUFS4 | ENSG00000164258 |
| Literature | NDUFS5 | ENSG00000168653 |
| Literature | NDUFS6 | ENSG00000145494 |
| Literature | NDUFS8 | ENSG00000110717 |
| Literature | NDUFV1 | ENSG00000167792 |
| Literature | NDUFV2 | ENSG00000178127 |
| Literature | NDUFV3 | ENSG00000160194 |
| Literature | MTND6 | ENSG00000198695 |
| Literature | MTND4L | ENSG00000212907 |
| Literature | MTND1 | ENSG00000198888 |
| Literature | MTND2 | ENSG00000198763 |
| Literature | MTND5 | ENSG00000198786 |
| Literature | MTND4 | ENSG00000198886 |
| Literature | MTND3 | ENSG00000198840 |
| Literature | NDUFA1 | ENSG00000125356 |
| Literature | NDUFS7 | ENSG00000115286 |
| Literature | NDUFS3 | ENSG00000213619 |
| Literature | NDUFB4 | ENSG00000065518 |
| Literature | NDUFB11 | ENSG00000147123 |

**Supplementary Table 3. Two-sample Mendelian randomization analysis of causal effects of metformin use, type 1 diabetes and type 2 diabetes on AA.**

| **Exposure** | **MR method** | **SNPs** | **Beta** | **Se** | **P** | **OR** | **OR_lci95** | **OR_uci95** |
| --- | --- | --- | --- | --- | --- | --- | --- | --- |
| Metformin use | MR Egger | 37 | 3.052950768 | 8.60641152 | 0.7249206 | 21.17774292 | 1.00E-06 | 448544003.4 |
| Metformin use | Weighted median | 37 | -5.538301305 | 3.05107619 | 0.069493794 | 0.003933202 | 9.95E-06 | 1.555517096 |
| Metformin use | Inverse variance weighted | 37 | -5.322799359 | 2.144226277 | 0.013050547 | 0.004879076 | 7.30E-05 | 0.326241995 |
| Metformin use | Simple mode | 37 | -9.086489598 | 5.962365548 | 0.136252691 | 0.000113185 | 9.52E-10 | 13.46033047 |
| Metformin use | Weighted mode | 37 | -8.132296176 | 5.511894804 | 0.148796074 | 0.000293893 | 5.98E-09 | 14.45467136 |
| Type 1 diabetes | MR Egger | 17 | 0.012509023 | 0.042134274 | 0.77062602 | 1.012587588 | 0.932324681 | 1.099760248 |
| Type 1 diabetes | Weighted median | 17 | -0.043870207 | 0.03329106 | 0.187578167 | 0.957078172 | 0.896622203 | 1.021610466 |
| Type 1 diabetes | Inverse variance weighted | 17 | -0.038232183 | 0.026185146 | 0.144270446 | 0.962489441 | 0.914337913 | 1.013176761 |
| Type 1 diabetes | Simple mode | 17 | -0.083055944 | 0.065256703 | 0.221288498 | 0.92029966 | 0.809807202 | 1.045868033 |
| Type 1 diabetes | Weighted mode | 17 | -0.031236522 | 0.030766106 | 0.325075629 | 0.969246298 | 0.912526557 | 1.029491557 |
| Type 2 diabetes | MR Egger | 28 | -0.299462988 | 0.1111254 | 0.012177028 | 0.741216156 | 0.596146539 | 0.921587821 |
| Type 2 diabetes | Weighted median | 28 | -0.143911619 | 0.076566804 | 0.060168574 | 0.86596428 | 0.745289493 | 1.006178325 |
| Type 2 diabetes | Inverse variance weighted | 28 | -0.105102164 | 0.052875675 | 0.0468424 | 0.900232546 | 0.811607453 | 0.998535233 |
| Type 2 diabetes | Simple mode | 28 | -0.174207571 | 0.125139031 | 0.175249903 | 0.840122494 | 0.657388525 | 1.073650936 |
| Type 2 diabetes | Weighted mode | 28 | -0.163049606 | 0.094877869 | 0.097149853 | 0.849549045 | 0.705385915 | 1.023175491 |

**Supplementary Table 4**. **Independent instruments for two-sample MR analysis: harmonized data.**

| **Exposure** | **SNP** | **Chr** | **Pos** | **EA** | **OA** | **EAF** | **F** | **SNP-Exposure Association** | | | **SNP-AA Association** | | |
| --- | --- | --- | --- | --- | --- | --- | --- | --- | --- | --- | --- | --- | --- |
|  |  |  |  |  |  |  |  | Beta | Se | *P* | Beta | Se | *P* |
| Metformin use | rs10001190 | 4 | 6284633 | G | A | 0.632112 | 61.73519549 | 0.0026373 | 0.000335655 | 3.90E-15 | -0.0133 | 0.0291 | 0.6474 |
| Metformin use | rs10195252 | 2 | 165513091 | C | T | 0.405055 | 33.81789047 | -0.00191685 | 0.000329621 | 6.10E-09 | 0.0443 | 0.0293 | 0.1304 |
| Metformin use | rs10420309 | 19 | 46150182 | G | A | 0.437532 | 33.4368981 | -0.00189448 | 0.000327625 | 7.40E-09 | 0.0476 | 0.0286 | 0.0960793 |
| Metformin use | rs10965246 | 9 | 22132698 | C | T | 0.176686 | 102.4984437 | -0.00429996 | 0.000424723 | 4.30E-24 | 0.021 | 0.0391 | 0.5921 |
| Metformin use | rs11257655 | 10 | 12307894 | T | C | 0.208209 | 47.1682845 | 0.00273499 | 0.000398227 | 6.50E-12 | -0.0401 | 0.032 | 0.2103 |
| Metformin use | rs11658063 | 17 | 36103872 | G | C | 0.602828 | 63.07109823 | -0.0026474 | 0.000333353 | 2.00E-15 | -0.0261 | 0.0309 | 0.3979 |
| Metformin use | rs11708067 | 3 | 123065778 | G | A | 0.242368 | 32.94766208 | -0.00216544 | 0.000377254 | 9.50E-09 | -0.0161 | 0.0372 | 0.664899 |
| Metformin use | rs1215468 | 13 | 80707429 | G | A | 0.291405 | 66.99346099 | -0.00292874 | 0.00035782 | 2.70E-16 | 3.00E-04 | 0.0307 | 0.9929 |
| Metformin use | rs13266634 | 8 | 118184783 | T | C | 0.309592 | 52.74462392 | -0.00254288 | 0.000350136 | 3.80E-13 | 0.0029 | 0.0289 | 0.9206 |
| Metformin use | rs1496653 | 3 | 23454790 | G | A | 0.203441 | 52.8812319 | -0.00291936 | 0.000401455 | 3.50E-13 | 0.038 | 0.0302 | 0.2088 |
| Metformin use | rs1613295 | 10 | 80954789 | G | T | 0.578289 | 53.68247685 | 0.00240145 | 0.000327761 | 2.40E-13 | -0.0069 | 0.029 | 0.8122 |
| Metformin use | rs17036160 | 3 | 12329783 | T | C | 0.117453 | 38.79153567 | -0.00313639 | 0.000503572 | 4.70E-10 | -0.0443 | 0.0372 | 0.2339 |
| Metformin use | rs17250977 | 5 | 14753745 | G | A | 0.040221 | 31.02535276 | 0.00458709 | 0.000823529 | 2.50E-08 | -0.1439 | 0.0987 | 0.1447 |
| Metformin use | rs17513135 | 1 | 40035686 | T | C | 0.227468 | 34.76479383 | 0.0022746 | 0.000385776 | 3.70E-09 | 0.0031 | 0.034 | 0.9265 |
| Metformin use | rs1800961 | 20 | 43042364 | T | C | 0.030964 | 33.04536264 | 0.00536609 | 0.000933475 | 9.00E-09 | 0.1418 | 0.0668 | 0.0336403 |
| Metformin use | rs2009222 | 19 | 13034543 | C | T | 0.632367 | 32.13917398 | 0.00189842 | 0.000334869 | 1.40E-08 | -0.0307 | 0.0305 | 0.3147 |
| Metformin use | rs2796441 | 9 | 84308948 | A | G | 0.418465 | 31.08989641 | -0.00182507 | 0.000327318 | 2.50E-08 | -0.0094 | 0.0284 | 0.7411 |
| Metformin use | rs34744311 | 10 | 94467287 | T | C | 0.377322 | 72.56942325 | -0.00284808 | 0.00033433 | 1.60E-17 | 0.0157 | 0.0287 | 0.5836 |
| Metformin use | rs4686471 | 3 | 187740899 | C | T | 0.610082 | 32.16554861 | 0.0018892 | 0.000333106 | 1.40E-08 | -0.0057 | 0.0288 | 0.842 |
| Metformin use | rs4752792 | 11 | 47815702 | A | G | 0.544491 | 41.58554008 | 0.00209176 | 0.00032437 | 1.10E-10 | -0.0344 | 0.0294 | 0.2418 |
| Metformin use | rs4932264 | 15 | 90422986 | C | T | 0.729643 | 36.94925556 | -0.0022175 | 0.000364805 | 1.20E-09 | -0.0412 | 0.0328 | 0.2093 |
| Metformin use | rs62106258 | 2 | 417167 | C | T | 0.04854 | 30.9532236 | -0.00418623 | 0.000752437 | 2.60E-08 | 0.1084 | 0.1002 | 0.2791 |
| Metformin use | rs67232546 | 11 | 128398938 | T | C | 0.212505 | 32.62157615 | 0.00227404 | 0.000398149 | 1.10E-08 | -0.0161 | 0.0393 | 0.6814 |
| Metformin use | rs6769511 | 3 | 185530290 | C | T | 0.315782 | 83.60269029 | 0.00318225 | 0.000348036 | 6.00E-20 | -0.0395 | 0.0304 | 0.194 |
| Metformin use | rs7018475 | 9 | 22137685 | G | T | 0.257503 | 54.16880433 | 0.00272318 | 0.00037 | 1.80E-13 | -0.0374 | 0.0314 | 0.2344 |
| Metformin use | rs7177055 | 15 | 77832762 | A | G | 0.717474 | 39.28866867 | 0.00224939 | 0.000358865 | 3.70E-10 | -0.0358 | 0.0304 | 0.2393 |
| Metformin use | rs72802357 | 16 | 75243142 | T | C | 0.078087 | 44.54955654 | -0.00404659 | 0.000606272 | 2.50E-11 | 0.1077 | 0.0519 | 0.0377503 |
| Metformin use | rs73188924 | 22 | 50788567 | A | C | 0.224797 | 31.1091987 | 0.00217376 | 0.000389733 | 2.40E-08 | -0.0733 | 0.0359 | 0.0414897 |
| Metformin use | rs7376543 | 4 | 49310408 | G | T | 0.68871 | 35.65491448 | -0.00232207 | 0.00038888 | 2.40E-09 | 0.0156 | 0.0341 | 0.6475 |
| Metformin use | rs74567345 | 5 | 102301358 | C | T | 0.051008 | 56.32073875 | 0.0056312 | 0.000750355 | 6.20E-14 | -0.0355 | 0.0598 | 0.552901 |
| Metformin use | rs7482891 | 11 | 2197112 | G | A | 0.622096 | 42.30375764 | -0.00217361 | 0.000334189 | 7.80E-11 | -0.0178 | 0.0296 | 0.5479 |
| Metformin use | rs76550717 | 11 | 72428172 | G | A | 0.159023 | 39.36548692 | -0.00278832 | 0.000444411 | 3.50E-10 | -0.0167 | 0.0328 | 0.6108 |
| Metformin use | rs76675804 | 2 | 43611883 | C | T | 0.100098 | 60.15124163 | -0.00418436 | 0.000539519 | 8.80E-15 | -0.0769 | 0.0654 | 0.2395 |
| Metformin use | rs780093 | 2 | 27742603 | C | T | 0.615229 | 38.62203695 | 0.00206338 | 0.000332018 | 5.10E-10 | -0.0227 | 0.0294 | 0.4397 |
| Metformin use | rs849142 | 7 | 28185891 | C | T | 0.505093 | 54.91427422 | -0.00239601 | 0.00032333 | 1.30E-13 | 0.0346 | 0.0281 | 0.218 |
| Metformin use | rs947791 | 11 | 65302893 | A | G | 0.217618 | 34.01578906 | 0.00228897 | 0.000392464 | 5.50E-09 | 0.0411 | 0.0492 | 0.4035 |
| Metformin use | rs9957264 | 18 | 56881633 | A | C | 0.166478 | 36.42313317 | -0.00262705 | 0.000435291 | 1.60E-09 | 0.0356 | 0.0327 | 0.2749 |
| Type 1 diabetes | rs114311872 | 6 | 32812736 | T | C | 0.04916 | 90.86076069 | -0.4604 | 0.0483 | 1.42E-21 | -0.0306 | 0.0644 | 0.6342 |
| Type 1 diabetes | rs115204205 | 2 | 60634304 | C | T | 0.03088 | 37.515625 | 0.3528 | 0.0576 | 9.34E-10 | -0.1578 | 0.0818 | 0.0538096 |
| Type 1 diabetes | rs13716 | 6 | 31604010 | G | C | 0.28 | 241.154437 | 0.3463 | 0.0223 | 1.32E-54 | -0.0769 | 0.0312 | 0.01378 |
| Type 1 diabetes | rs144536316 | 6 | 27434094 | A | G | 0.02952 | 69.16008753 | 0.4707 | 0.0566 | 9.40E-17 | -0.0145 | 0.084 | 0.8631 |
| Type 1 diabetes | rs1611236 | 6 | 29748690 | A | G | 0.2234 | 43.66782174 | -0.1619 | 0.0245 | 3.60E-11 | 0.0134 | 0.0348 | 0.7001 |
| Type 1 diabetes | rs193474 | 22 | 30449094 | G | A | 0.6833 | 31.77342916 | -0.1195 | 0.0212 | 1.63E-08 | -0.0046 | 0.0303 | 0.8788 |
| Type 1 diabetes | rs1980496 | 6 | 32340070 | T | C | 0.2981 | 1353.653869 | 0.8315 | 0.0226 | 1.00E-200 | 0.0194 | 0.0324 | 0.549201 |
| Type 1 diabetes | rs2844696 | 6 | 30933371 | T | C | 0.02316 | 126.063732 | 0.759 | 0.0676 | 3.10E-29 | 0.1812 | 0.0964 | 0.0600302 |
| Type 1 diabetes | rs41291770 | 6 | 29594265 | C | T | 0.06105 | 81.40499937 | -0.4015 | 0.0445 | 1.79E-19 | 0.065 | 0.0583 | 0.2651 |
| Type 1 diabetes | rs62146643 | 2 | 59402928 | T | C | 0.0283 | 32.62571933 | 0.341 | 0.0597 | 1.13E-08 | 0.0905 | 0.0847 | 0.2854 |
| Type 1 diabetes | rs6679677 | 1 | 114303808 | A | C | 0.1472 | 205.1724902 | 0.4025 | 0.0281 | 1.21E-46 | -0.0263 | 0.0397 | 0.508401 |
| Type 1 diabetes | rs705699 | 12 | 56384804 | A | G | 0.3857 | 37.97716518 | 0.1251 | 0.0203 | 7.49E-10 | -0.015 | 0.0289 | 0.6039 |
| Type 1 diabetes | rs74203920 | 21 | 45714294 | T | C | 0.03727 | 34.63130478 | 0.3066 | 0.0521 | 3.85E-09 | 0.0441 | 0.0746 | 0.5543 |
| Type 1 diabetes | rs7903146 | 10 | 114758349 | T | C | 0.1947 | 38.69939195 | 0.1549 | 0.0249 | 5.10E-10 | -0.0559 | 0.0351 | 0.1108 |
| Type 1 diabetes | rs9264277 | 6 | 31224667 | C | T | 0.7297 | 80.44343723 | 0.2027 | 0.0226 | 3.17E-19 | -0.0325 | 0.0318 | 0.3067 |
| Type 1 diabetes | rs9275468 | 6 | 32672412 | A | G | 0.4119 | 1380.337426 | 0.8991 | 0.0242 | 1.00E-200 | -0.0579 | 0.0345 | 0.0925806 |
| Type 1 diabetes | rs9468618 | 6 | 29750776 | T | C | 0.05595 | 54.33795918 | -0.3354 | 0.0455 | 1.79E-13 | 0.0353 | 0.0603 | 0.5583 |
| Type 2 diabetes | rs1046317 | 4 | 6304242 | C | T | 0.6099 | 40.52177545 | 0.0853 | 0.0134 | 2.21E-10 | -0.0154 | 0.0288 | 0.5919 |
| Type 2 diabetes | rs10830963 | 11 | 92708710 | G | C | 0.3548 | 40.41989451 | 0.0871 | 0.0137 | 1.95E-10 | -0.0125 | 0.0294 | 0.6708 |
| Type 2 diabetes | rs10882099 | 10 | 94460650 | C | T | 0.4774 | 30.04044053 | -0.0718 | 0.0131 | 4.36E-08 | 0.0243 | 0.0281 | 0.3876 |
| Type 2 diabetes | rs10965246 | 9 | 22132698 | C | T | 0.1524 | 43.79112544 | -0.1211 | 0.0183 | 3.77E-11 | 0.021 | 0.0391 | 0.5921 |
| Type 2 diabetes | rs112108223 | 12 | 4271088 | A | G | 0.02278 | 36.85267215 | -0.275 | 0.0453 | 1.23E-09 | 0.0942 | 0.0951 | 0.3218 |
| Type 2 diabetes | rs11257658 | 10 | 12309268 | A | G | 0.2639 | 37.62875546 | 0.0914 | 0.0149 | 9.05E-10 | -0.0408 | 0.0321 | 0.2037 |
| Type 2 diabetes | rs11712037 | 3 | 12344730 | G | C | 0.1715 | 29.87187872 | -0.0951 | 0.0174 | 4.47E-08 | -0.0463 | 0.0372 | 0.2134 |
| Type 2 diabetes | rs1815311 | 6 | 7245458 | G | A | 0.4047 | 32.93389396 | 0.0769 | 0.0134 | 8.93E-09 | 0.0464 | 0.0287 | 0.1059 |
| Type 2 diabetes | rs2237897 | 11 | 2858546 | T | C | 0.08193 | 46.60036883 | -0.1652 | 0.0242 | 8.67E-12 | 0.0188 | 0.0515 | 0.7149 |
| Type 2 diabetes | rs2254021 | 17 | 3935310 | A | T | 0.3951 | 30.41440187 | 0.0739 | 0.0134 | 3.32E-08 | 0.0167 | 0.0287 | 0.5597 |
| Type 2 diabetes | rs2394972 | 6 | 31311760 | G | A | 0.7391 | 39.1254448 | 0.0932 | 0.0149 | 3.89E-10 | 0.0471 | 0.0319 | 0.1403 |
| Type 2 diabetes | rs28624681 | 9 | 139237902 | C | T | 0.7068 | 39.84765625 | 0.0909 | 0.0144 | 3.16E-10 | 0.0091 | 0.0309 | 0.768 |
| Type 2 diabetes | rs3104368 | 6 | 32602561 | T | C | 0.1525 | 163.1488386 | 0.2363 | 0.0185 | 1.59E-37 | -0.0176 | 0.0396 | 0.657099 |
| Type 2 diabetes | rs34298980 | 6 | 40409243 | C | T | 0.3446 | 33.57421659 | -0.0817 | 0.0141 | 7.51E-09 | 0.0129 | 0.0303 | 0.6709 |
| Type 2 diabetes | rs34872471 | 10 | 114754071 | C | T | 0.1995 | 273.842684 | 0.2747 | 0.0166 | 2.56E-61 | -0.0542 | 0.035 | 0.1215 |
| Type 2 diabetes | rs3887925 | 3 | 186665645 | T | C | 0.463 | 36.5517161 | 0.0792 | 0.0131 | 1.73E-09 | -0.0142 | 0.0282 | 0.613901 |
| Type 2 diabetes | rs45551238 | 20 | 57607363 | T | C | 0.05033 | 51.73652228 | -0.2201 | 0.0306 | 6.12E-13 | 2.00E-04 | 0.0648 | 0.9973 |
| Type 2 diabetes | rs55993634 | 16 | 75236763 | G | C | 0.08801 | 38.24865074 | -0.1441 | 0.0233 | 6.74E-10 | 0.0648 | 0.0498 | 0.1935 |
| Type 2 diabetes | rs56348580 | 12 | 121432117 | C | G | 0.2836 | 31.85288047 | -0.0824 | 0.0146 | 1.63E-08 | 0.0092 | 0.0312 | 0.7692 |
| Type 2 diabetes | rs590015 | 6 | 139824992 | A | G | 0.5027 | 30.71359478 | 0.0726 | 0.0131 | 2.79E-08 | 0.0369 | 0.028 | 0.1886 |
| Type 2 diabetes | rs7018475 | 9 | 22137685 | G | T | 0.2782 | 55.22729405 | 0.1085 | 0.0146 | 1.16E-13 | -0.0374 | 0.0314 | 0.2344 |
| Type 2 diabetes | rs7109575 | 11 | 72463435 | A | G | 0.2382 | 35.76661326 | -0.0921 | 0.0154 | 2.36E-09 | -0.0226 | 0.0331 | 0.494899 |
| Type 2 diabetes | rs73193383 | 4 | 3392274 | T | C | 0.07966 | 30.18213687 | 0.1335 | 0.0243 | 3.92E-08 | 0.0108 | 0.0518 | 0.8347 |
| Type 2 diabetes | rs7633675 | 3 | 185510613 | G | T | 0.3067 | 44.72858508 | 0.0943 | 0.0141 | 2.54E-11 | -0.0362 | 0.0304 | 0.2328 |
| Type 2 diabetes | rs76895963 | 12 | 4384844 | G | T | 0.03183 | 91.903545 | -0.3873 | 0.0404 | 8.20E-22 | 0.1706 | 0.0842 | 0.0427602 |
| Type 2 diabetes | rs77655131 | 7 | 102086552 | T | C | 0.1828 | 31.49190311 | 0.0954 | 0.017 | 2.03E-08 | 0.034 | 0.0365 | 0.3509 |
| Type 2 diabetes | rs78470967 | 12 | 4521511 | A | T | 0.0399 | 33.71488033 | -0.198 | 0.0341 | 6.67E-09 | 0.0288 | 0.0727 | 0.6916 |
| Type 2 diabetes | rs9273401 | 6 | 32627129 | G | A | 0.1234 | 196.4140115 | 0.2845 | 0.0203 | 1.79E-44 | -0.0374 | 0.0437 | 0.3921 |

**Supplementary Table 5**. **Sensitivity analysis of two-sample MR analysis.**

| **Exposure** | **MR-PRESSO** | **Heterogeneity**  **(Inverse variance weighted)** | | **pleiotropy analysis** | | | **steiger test** |  |
| --- | --- | --- | --- | --- | --- | --- | --- | --- |
|  | global test p-value | Q | Q_pval | MR-Egger intercept | MR-Egger SE | MR-Egger P-value | correct_causal_direction | steiger_pval |
| Metformin | 0.4566 | 36.72233047 | 0.435218021 | -0.022583535 | 0.022473925 | 0.321853056 | TRUE | 3.00E-69 |
| Type 1 diabetes | 0.1826 | 21.44074281 | 0.162184283 | -0.028144508 | 0.018724931 | 0.153585913 | TRUE | 5.93E-295 |
| Type 2 diabetes | 0.7075 | 22.80596254 | 0.695391631 | 0.027767218 | 0.013963488 | 0.057371651 | TRUE | 3.19E-140 |

**Supplementary Table 6.** **Causal relationships of metformin use on AA adjusted for type 1 and 2 diabetes estimated by multivariable MR.**

| **Exposure** | **SNPs** | **MVMR-IVW** | | | | **MVMR-Egger** | | | |
| --- | --- | --- | --- | --- | --- | --- | --- | --- | --- |
|  |  | OR | OR_lci95 | OR_uci95 | *P* | OR | OR_lci95 | OR_uci95 | *P* |
| Metformin use | 80 | 0.000111209 | 3.56E-08 | 0.3472719 | 0.02658301 | 0.000133547 | 3.97E-08 | 0.4491168 | 0.03130579 |
| Type 1 diabetes | 80 | 0.957569945 | 9.05E-01 | 1.0130456 | 0.13132714 | 0.96168218 | 9.06E-01 | 1.0203285 | 0.19578941 |
| Type 2 diabetes | 80 | 1.125095226 | 8.46E-01 | 1.4956722 | 0.41712734 | 1.139464302 | 8.52E-01 | 1.5238843 | 0.37872954 |
| Heterogeneity (Inverse variance weighted) |  | Q=98.9968 | | | 0.0465 | Q=98.6921 | | | 0.0412 |
| Intercept |  |  |  |  |  | 0.9970053 | 0.9849795 | 1.009178 | 0.6280968 |

**Supplementary Table 7.** **The results of 5 metformin target genes in blood and aortic aneurysm of Mendelian randomization in eQTLGen.**

| **Exposure** | **MR method** | **SNPs** | **Beta** | **Se** | ***P*** | **OR** | **OR_lci95** | **OR_uci95** |
| --- | --- | --- | --- | --- | --- | --- | --- | --- |
| PRKAA1 | MR Egger | 12 | -0.027291576 | 0.447367345 | 0.952557375 | 0.973077474 | 0.404893353 | 2.338590553 |
| PRKAA1 | Weighted median | 12 | -0.475837755 | 0.145329374 | 0.001059608 | 0.621364287 | 0.467346966 | 0.826139048 |
| PRKAA1 | Inverse variance weighted | 12 | -0.432290826 | 0.111618 | 0.000107526 | 0.649020597 | 0.521491603 | 0.807736372 |
| PRKAA1 | Simple mode | 12 | -0.463071515 | 0.20349009 | 0.043870383 | 0.629347623 | 0.422353731 | 0.937788403 |
| PRKAA1 | Weighted mode | 12 | -0.46983973 | 0.156899104 | 0.012198431 | 0.625102445 | 0.459616894 | 0.850171244 |
| HMGB3 | Wald ratio | 1 | -2.458824102 | 0.5444381 | 6.29413E-06 | 0.085535473 | 0.029424642 | 0.248645914 |
| CYB5B | MR Egger | 39 | 0.209091849 | 0.123489679 | 0.09882343 | 1.232558203 | 0.967588948 | 1.570087924 |
| CYB5B | Weighted median | 39 | 0.23491244 | 0.070012335 | 0.000792787 | 1.264798018 | 1.102619004 | 1.450831176 |
| CYB5B | Inverse variance weighted | 39 | 0.29755742 | 0.057610666 | 2.40476E-07 | 1.346565692 | 1.202785977 | 1.507532677 |
| CYB5B | Simple mode | 39 | 0.215560747 | 0.112561457 | 0.063035698 | 1.240557341 | 0.99495307 | 1.546789051 |
| CYB5B | Weighted mode | 39 | 0.209324884 | 0.085913552 | 0.01962788 | 1.232845465 | 1.04178368 | 1.458947735 |
| NDUFA6 | MR Egger | 78 | 0.119498999 | 0.031575709 | 0.000305406 | 1.126932116 | 1.059302432 | 1.198879522 |
| NDUFA6 | Weighted median | 78 | 0.118251342 | 0.030236107 | 9.19408E-05 | 1.125526968 | 1.06076312 | 1.19424491 |
| NDUFA6 | Inverse variance weighted | 78 | 0.10845231 | 0.022654957 | 1.6918E-06 | 1.114551755 | 1.066144207 | 1.165157214 |
| NDUFA6 | Simple mode | 78 | 0.091432439 | 0.049142429 | 0.06662425 | 1.095742745 | 0.995125199 | 1.206533775 |
| NDUFA6 | Weighted mode | 78 | 0.112196779 | 0.029023157 | 0.000229724 | 1.118732982 | 1.056869663 | 1.184217439 |
| NDUFAF3 | MR Egger | 47 | -0.620015601 | 0.139676698 | 5.79884E-05 | 0.537936045 | 0.409105615 | 0.707336146 |
| NDUFAF3 | Weighted median | 47 | -0.48690502 | 0.074635235 | 6.85501E-11 | 0.614525398 | 0.530895497 | 0.71132919 |
| NDUFAF3 | Inverse variance weighted | 47 | -0.358689299 | 0.053422782 | 1.89148E-11 | 0.69859137 | 0.629142214 | 0.775706814 |
| NDUFAF3 | Simple mode | 47 | -0.555353775 | 0.111711524 | 9.67781E-06 | 0.573869204 | 0.461022531 | 0.714337894 |
| NDUFAF3 | Weighted mode | 47 | -0.531059553 | 0.081375819 | 4.69527E-08 | 0.587981642 | 0.501297191 | 0.689655593 |

**Supplementary Table 8**. **The results of 2 metformin target genes in blood and aortic aneurysm of Mendelian randomization in GTEx.**

| **Exposure** | **MR method** | **SNPs** | **Beta** | **Se** | **P** | **OR** | **OR_lci95** | **OR_uci95** |
| --- | --- | --- | --- | --- | --- | --- | --- | --- |
| CYB5B | Wald ratio | 1 | 0.569685124 | 0.224060258 | 0.011004585 | 1.767710354 | 1.139428725 | 2.742426821 |
| NDUFA6 | MR Egger | 12 | 0.211745987 | 0.081961431 | 0.027252961 | 1.235833927 | 1.052429796 | 1.451199406 |
| NDUFA6 | Weighted median | 12 | 0.211758573 | 0.072534256 | 0.003506738 | 1.235849482 | 1.072070078 | 1.424649352 |
| NDUFA6 | Inverse variance weighted | 12 | 0.258473653 | 0.057423963 | 6.76E-06 | 1.294952031 | 1.157106723 | 1.449218753 |
| NDUFA6 | Simple mode | 12 | 0.195495654 | 0.101501786 | 0.080326103 | 1.215913509 | 0.99655809 | 1.483551915 |
| NDUFA6 | Weighted mode | 12 | 0.210011065 | 0.08574043 | 0.032281154 | 1.23369171 | 1.042852579 | 1.459453874 |

**Supplementary Table 9**. **The results of heterogeneity, pleiotropy analysis and steiger test in eQTLGen.**

| **Exposure** | **Heterogeneity (Inverse variance weighted)** | | **Pleiotropy analysis** | | | **Steiger test** | |
| --- | --- | --- | --- | --- | --- | --- | --- |
|  | Q | Q_pval | MR-Egger intercept | MR-Egger SE | MR-Egger P-value | correct_causal_direction | steiger_pval |
| PRKAA1 | 5.91693942 | 0.878848369 | -0.045505544 | 0.048676337 | 0.371885508 | TRUE | 0.272672416 |
| HMGB3 | - | - | - | - | - | FALSE | 0.052515918 |
| CYB5B | 58.86492383 | 0.016536268 | 0.014280883 | 0.017610191 | 0.422583604 | TRUE | 0.005674209 |
| NDUFA6 | 78.47853072 | 0.431695673 | -0.003854229 | 0.007634239 | 0.615117382 | TRUE | 6.07E-222 |
| NDUFAF3 | 58.29835417 | 0.105442265 | 0.034141994 | 0.016950872 | 0.049992379 | TRUE | 3.96E-06 |

**Supplementary Table 10. The results of heterogeneity, pleiotropy analysis and steiger test in GTEx.**

| **Exposure** | **Heterogeneity (Inverse variance weighted)** | | **Pleiotropy analysis** | | | **Steiger test** | |
| --- | --- | --- | --- | --- | --- | --- | --- |
|  | Q | Q_pval | MR-Egger intercept | MR-Egger SE | MR-Egger P-value | correct_causal_direction | steiger_pval |
| CYB5B | - | - | - | - | - | TRUE | 9.47E-05 |
| NDUFA6 | 5.402160857 | 0.910138392 | 0.018615082 | 0.023297771 | 0.442850024 | TRUE | 0 |

**Supplementary Table 11. The results of NDUFA6 and aortic aneurysm of Mendelian randomization in 5 tissues in GTEx.**

| **Tissue** | **Exposure** | **MR method** | **SNPs** | **Beta** | **Se** | ***P*** | **OR** | **OR_lci95** | **OR_uci95** |
| --- | --- | --- | --- | --- | --- | --- | --- | --- | --- |
| aorta | NDUFA6 | MR Egger | 9 | 0.120277683 | 0.049370858 | 0.045008653 | 1.127809981 | 1.023789359 | 1.242399467 |
| aorta | NDUFA6 | Weighted median | 9 | 0.103899181 | 0.036052186 | 0.003952717 | 1.109488592 | 1.033795413 | 1.190723928 |
| aorta | NDUFA6 | Inverse variance weighted | 9 | 0.123033776 | 0.028943815 | 2.13E-05 | 1.130922618 | 1.068551398 | 1.196934439 |
| aorta | NDUFA6 | Simple mode | 9 | 0.094638175 | 0.052581698 | 0.109581293 | 1.099261044 | 0.991613412 | 1.218594693 |
| aorta | NDUFA6 | Weighted mode | 9 | 0.098361041 | 0.045629452 | 0.063216588 | 1.103361072 | 1.008967282 | 1.206585858 |
| tibial artery | NDUFA6 | MR Egger | 14 | 0.137463313 | 0.041846624 | 0.006520259 | 1.147359612 | 1.057009749 | 1.245432296 |
| tibial artery | NDUFA6 | Weighted median | 14 | 0.115246992 | 0.037635233 | 0.002197092 | 1.122150565 | 1.042354331 | 1.208055508 |
| tibial artery | NDUFA6 | Inverse variance weighted | 14 | 0.126878773 | 0.029653819 | 1.88E-05 | 1.135279383 | 1.071176191 | 1.203218749 |
| tibial artery | NDUFA6 | Simple mode | 14 | 0.099321206 | 0.053813445 | 0.087833806 | 1.10442099 | 0.993865746 | 1.227274134 |
| tibial artery | NDUFA6 | Weighted mode | 14 | 0.11061645 | 0.042638039 | 0.022244653 | 1.116966412 | 1.027414954 | 1.214323348 |
| coronary artery | NDUFA6 | MR Egger | 4 | -0.128633968 | 0.19414809 | 0.57575338 | 0.879295757 | 0.600997668 | 1.286462611 |
| coronary artery | NDUFA6 | Weighted median | 4 | 0.158835428 | 0.066540463 | 0.016984171 | 1.172145028 | 1.028823709 | 1.335431867 |
| coronary artery | NDUFA6 | Inverse variance weighted | 4 | 0.191274737 | 0.052140623 | 0.000244035 | 1.210792056 | 1.09316714 | 1.341073426 |
| coronary artery | NDUFA6 | Simple mode | 4 | 0.160481533 | 0.073861141 | 0.118150804 | 1.174076091 | 1.015837805 | 1.356963345 |
| coronary artery | NDUFA6 | Weighted mode | 4 | 0.154852314 | 0.064118282 | 0.094583007 | 1.167485527 | 1.029610402 | 1.323823508 |
| visceral omental adipose | NDUFA6 | MR Egger | 13 | 0.181971077 | 0.067659668 | 0.021047716 | 1.199579498 | 1.05059653 | 1.369689439 |
| visceral omental adipose | NDUFA6 | Weighted median | 13 | 0.16540549 | 0.054316669 | 0.002325204 | 1.179871448 | 1.060716707 | 1.312411338 |
| visceral omental adipose | NDUFA6 | Inverse variance weighted | 13 | 0.178960466 | 0.04068719 | 1.09E-05 | 1.195973462 | 1.104302125 | 1.295254703 |
| visceral omental adipose | NDUFA6 | Simple mode | 13 | 0.150333419 | 0.07156982 | 0.057492425 | 1.162221685 | 1.010107309 | 1.337243313 |
| visceral omental adipose | NDUFA6 | Weighted mode | 13 | 0.159780986 | 0.065817515 | 0.031869854 | 1.173253884 | 1.031257217 | 1.334802466 |
| tibial nerve | NDUFA6 | MR Egger | 12 | 0.143991995 | 0.045627031 | 0.010230739 | 1.154874864 | 1.056079023 | 1.262913024 |
| tibial nerve | NDUFA6 | Weighted median | 12 | 0.119748982 | 0.04023085 | 0.002915152 | 1.127213865 | 1.041744297 | 1.219695756 |
| tibial nerve | NDUFA6 | Inverse variance weighted | 12 | 0.134325824 | 0.032637836 | 3.86E-05 | 1.143765425 | 1.072889702 | 1.21932324 |
| tibial nerve | NDUFA6 | Simple mode | 12 | 0.114480961 | 0.055112301 | 0.061994029 | 1.121291293 | 1.006481768 | 1.249197157 |
| tibial nerve | NDUFA6 | Weighted mode | 12 | 0.116965314 | 0.045571626 | 0.026207459 | 1.124080439 | 1.028030593 | 1.229104311 |

**Supplementary Table 12. The results of heterogeneity, pleiotropy analysis and steiger test in 5 tissues.**

| **Tissue** | **Exposure** | **Heterogeneity**  **(Inverse variance weighted)** | | **Pleiotropy analysis** | | | **Steiger test** | |
| --- | --- | --- | --- | --- | --- | --- | --- | --- |
|  |  | Q | Q_pval | MR-Egger intercept | MR-Egger SE | MR-Egger P-value | correct_causal_direction | steiger_pval |
| aorta | NDUFA6 | 4.064761678 | 0.851233646 | 0.002621234 | 0.038039628 | 0.946989963 | TRUE | 0 |
| tibial artery | NDUFA6 | 5.07556902 | 0.973491681 | -0.006448991 | 0.017989784 | 0.726210971 | TRUE | 0 |
| coronary artery | NDUFA6 | 2.941803699 | 0.400685208 | 0.296935562 | 0.173585723 | 0.229283878 | TRUE | 0 |
| visceral omental adipose | NDUFA6 | 4.779773884 | 0.964932924 | -0.00151126 | 0.027136457 | 0.956586536 | TRUE | 0 |
| tibial nerve | NDUFA6 | 5.684593681 | 0.893558631 | -0.006780887 | 0.022366939 | 0.767976739 | TRUE | 0 |

**Supplementary Table 13. A detailed summary of genetic variants in eQTL-MR analysis: harmonized data.**

| **Data source** | **Tissue** | **Exposure** | **SNP** | **Chr** | **Pos** | **EA** | **OA** | **EAF** | **F** | **SNP-Exposure Association** | | | **SNP-AA Association** | | |
| --- | --- | --- | --- | --- | --- | --- | --- | --- | --- | --- | --- | --- | --- | --- | --- |
|  |  |  |  |  |  |  |  |  |  | Beta | Se | P | Beta | Se | P |
| eQTLGen | blood | PRKAA1 | rs143913574 | 5 | 40799030 | A | G | 0.0196087 | 17.82367869 | 0.181111 | 0.0428989 | 2.42359E-05 | 0.0364 | 0.0714 | 0.609901 |
| eQTLGen | blood | PRKAA1 | rs1505995 | 5 | 40609125 | G | C | 0.165724 | 24.76562927 | 0.0795868 | 0.0159925 | 6.47784E-07 | -0.0744 | 0.04 | 0.0625994 |
| eQTLGen | blood | PRKAA1 | rs1910015 | 5 | 40913845 | T | G | 0.182447 | 54.44804718 | 0.113494 | 0.0153809 | 1.59735E-13 | -0.0706 | 0.0356 | 0.0475204 |
| eQTLGen | blood | PRKAA1 | rs36012714 | 5 | 40805979 | A | G | 0.211083 | 26.27384707 | -0.0746896 | 0.0145713 | 2.96278E-07 | 0.0276 | 0.0366 | 0.4506 |
| eQTLGen | blood | PRKAA1 | rs3805487 | 5 | 40796033 | T | C | 0.276148 | 103.5488376 | 0.134969 | 0.0132636 | 2.54273E-24 | -0.0614 | 0.0312 | 0.0495199 |
| eQTLGen | blood | PRKAA1 | rs4957342 | 5 | 40780569 | C | T | 0.259475 | 82.16673127 | 0.12272 | 0.0135384 | 1.25055E-19 | -0.0609 | 0.0312 | 0.0511399 |
| eQTLGen | blood | PRKAA1 | rs62357603 | 5 | 40850283 | A | G | 0.202427 | 32.27826472 | -0.0840591 | 0.0147955 | 1.33601E-08 | 0.0407 | 0.0356 | 0.2526 |
| eQTLGen | blood | PRKAA1 | rs62360859 | 5 | 41109510 | A | C | 0.0153839 | 25.54500152 | -0.244196 | 0.0483154 | 4.32195E-07 | 0.1102 | 0.2046 | 0.59 |
| eQTLGen | blood | PRKAA1 | rs6703865 | 1 | 169550963 | A | G | 0.0761144 | 19.89230873 | -0.100032 | 0.0224283 | 8.18823E-06 | -0.0486 | 0.0686 | 0.4783 |
| eQTLGen | blood | PRKAA1 | rs74315832 | 5 | 40495997 | A | G | 0.103738 | 33.80140325 | 0.113346 | 0.0194957 | 6.10703E-09 | -0.0487 | 0.052 | 0.3484 |
| eQTLGen | blood | PRKAA1 | rs7716062 | 5 | 40951111 | A | T | 0.80191 | 27.48383401 | 0.0782108 | 0.0149186 | 1.58329E-07 | -0.0433 | 0.0353 | 0.2197 |
| eQTLGen | blood | PRKAA1 | rs80272243 | 5 | 40569806 | C | G | 0.0406137 | 16.58857696 | -0.122733 | 0.030134 | 4.64109E-05 | 0.0558 | 0.0765 | 0.466 |
| eQTLGen | blood | HMGB3 | rs740406 | 19 | 2232221 | G | A | 0.0798575 | 16.96044416 | 0.0903684 | 0.0219431 | 3.81839E-05 | -0.2222 | 0.0492 | 6.34103E-06 |
| eQTLGen | blood | CYB5B | rs11075725 | 16 | 69532023 | C | T | 0.41235 | 97.17034756 | 0.118776 | 0.0120493 | 6.36796E-23 | -0.0212 | 0.0288 | 0.4626 |
| eQTLGen | blood | CYB5B | rs111290442 | 16 | 69156345 | A | G | 0.030705 | 16.89117785 | -0.141704 | 0.0344788 | 3.95658E-05 | -0.0673 | 0.09 | 0.4545 |
| eQTLGen | blood | CYB5B | rs111579298 | 16 | 68576507 | T | C | 0.0323151 | 80.03270084 | -0.300249 | 0.033562 | 3.67959E-19 | -0.0079 | 0.0632 | 0.9001 |
| eQTLGen | blood | CYB5B | rs112512634 | 16 | 69532492 | T | C | 0.0735372 | 125.5952793 | -0.254413 | 0.0227014 | 3.76791E-29 | -0.0636 | 0.0634 | 0.3158 |
| eQTLGen | blood | CYB5B | rs11641710 | 16 | 69509506 | G | A | 0.733844 | 149.446993 | -0.163769 | 0.0133964 | 2.2914E-34 | -0.0853 | 0.0304 | 0.00504406 |
| eQTLGen | blood | CYB5B | rs117249563 | 16 | 69420673 | C | A | 0.0351146 | 47.50916126 | 0.222496 | 0.03228 | 5.47772E-12 | 0.0497 | 0.0733 | 0.498 |
| eQTLGen | blood | CYB5B | rs117274281 | 16 | 69477476 | A | G | 0.0391728 | 19.51508509 | -0.13543 | 0.030657 | 9.97103E-06 | 0.1617 | 0.078 | 0.0380496 |
| eQTLGen | blood | CYB5B | rs117300597 | 16 | 68805234 | T | C | 0.0318706 | 117.9502445 | -0.366459 | 0.0337424 | 1.77582E-27 | -0.0922 | 0.0551 | 0.0943996 |
| eQTLGen | blood | CYB5B | rs11859467 | 16 | 68800791 | T | C | 0.0229507 | 55.99522826 | -0.296828 | 0.039667 | 7.2644E-14 | -0.0165 | 0.072 | 0.8186 |
| eQTLGen | blood | CYB5B | rs11866219 | 16 | 69549749 | C | A | 0.602758 | 24.45603052 | -0.0600983 | 0.0121526 | 7.60659E-07 | -0.0245 | 0.0286 | 0.3914 |
| eQTLGen | blood | CYB5B | rs12051035 | 16 | 68734554 | C | T | 0.428311 | 38.24564943 | -0.0742828 | 0.0120115 | 6.2339E-10 | -0.0146 | 0.0283 | 0.6067 |
| eQTLGen | blood | CYB5B | rs12919044 | 16 | 69240509 | A | G | 0.410924 | 62.24143787 | 0.0952273 | 0.0120704 | 3.04159E-15 | 0.1048 | 0.0289 | 0.000291897 |
| eQTLGen | blood | CYB5B | rs12927073 | 16 | 68805641 | G | A | 0.852352 | 28.34404236 | -0.0892309 | 0.0167604 | 1.0162E-07 | 0.0098 | 0.0389 | 0.8015 |
| eQTLGen | blood | CYB5B | rs13015993 | 2 | 217625523 | A | G | 0.719968 | 16.63747942 | -0.0540345 | 0.0132473 | 4.52689E-05 | 0.057 | 0.029 | 0.0491405 |
| eQTLGen | blood | CYB5B | rs143437154 | 16 | 69084485 | T | C | 0.0281605 | 37.31765801 | -0.219488 | 0.0359297 | 1.00261E-09 | -0.0626 | 0.0816 | 0.4429 |
| eQTLGen | blood | CYB5B | rs146048597 | 16 | 68704853 | A | G | 0.132781 | 21.49517516 | -0.0812556 | 0.017526 | 3.54528E-06 | -0.0006 | 0.0384 | 0.9872 |
| eQTLGen | blood | CYB5B | rs150830331 | 16 | 69614785 | T | C | 0.0380193 | 52.70899762 | -0.225524 | 0.0310635 | 3.86545E-13 | -0.0543 | 0.0538 | 0.3129 |
| eQTLGen | blood | CYB5B | rs153050 | 16 | 69349738 | C | T | 0.632061 | 86.6425515 | -0.114529 | 0.0123041 | 1.30257E-20 | -0.095 | 0.0293 | 0.001187 |
| eQTLGen | blood | CYB5B | rs166134 | 16 | 69407915 | T | C | 0.9812 | 16.92583959 | -0.18018 | 0.0437957 | 3.88857E-05 | -0.1185 | 0.0931 | 0.203 |
| eQTLGen | blood | CYB5B | rs1812545 | 16 | 68990649 | A | G | 0.0829451 | 40.9443674 | -0.137886 | 0.0215488 | 1.56599E-10 | -0.1376 | 0.0497 | 0.005626 |
| eQTLGen | blood | CYB5B | rs28541473 | 16 | 69465496 | C | T | 0.19148 | 38.67466812 | 0.0939414 | 0.0151058 | 5.00968E-10 | 0.1099 | 0.039 | 0.00487001 |
| eQTLGen | blood | CYB5B | rs35828316 | 16 | 68993347 | A | G | 0.062735 | 38.64239961 | 0.152369 | 0.0245112 | 5.09343E-10 | 0.0094 | 0.0614 | 0.8777 |
| eQTLGen | blood | CYB5B | rs4783712 | 16 | 69538787 | C | T | 0.470262 | 26.30462491 | 0.061102 | 0.0119135 | 2.91588E-07 | -0.0278 | 0.0295 | 0.3465 |
| eQTLGen | blood | CYB5B | rs55811436 | 16 | 68510995 | C | T | 0.0489569 | 72.29424864 | -0.233927 | 0.0275124 | 1.85055E-17 | -0.0337 | 0.0532 | 0.5263 |
| eQTLGen | blood | CYB5B | rs58264433 | 16 | 69354682 | A | T | 0.12971 | 74.98819868 | -0.152993 | 0.0176675 | 4.73369E-18 | -0.0358 | 0.0421 | 0.3949 |
| eQTLGen | blood | CYB5B | rs62053264 | 16 | 69974773 | A | G | 0.065668 | 31.25796376 | -0.134189 | 0.0240014 | 2.25772E-08 | -0.0831 | 0.0403 | 0.0390499 |
| eQTLGen | blood | CYB5B | rs6499215 | 16 | 69112398 | C | A | 0.214138 | 35.43340154 | -0.0862555 | 0.0144904 | 2.63919E-09 | -0.084 | 0.0339 | 0.01333 |
| eQTLGen | blood | CYB5B | rs6499237 | 16 | 69654245 | T | G | 0.266759 | 20.27235927 | 0.0605485 | 0.0134478 | 6.71893E-06 | 0.0317 | 0.0319 | 0.3213 |
| eQTLGen | blood | CYB5B | rs689457 | 16 | 69761176 | T | C | 0.103399 | 34.85481322 | -0.115261 | 0.0195232 | 3.54789E-09 | -0.0426 | 0.0376 | 0.2568 |
| eQTLGen | blood | CYB5B | rs72789225 | 16 | 69107204 | C | A | 0.0412057 | 78.35268576 | 0.264319 | 0.0298608 | 8.6278E-19 | 0.0399 | 0.0688 | 0.561399 |
| eQTLGen | blood | CYB5B | rs72789256 | 16 | 69231250 | A | G | 0.0406411 | 92.55757227 | 0.28904 | 0.0300436 | 6.54485E-22 | 0.0421 | 0.0684 | 0.5384 |
| eQTLGen | blood | CYB5B | rs72795299 | 16 | 69385426 | A | G | 0.0400635 | 84.63815142 | 0.278377 | 0.0302587 | 3.58592E-20 | 0.0468 | 0.0693 | 0.4996 |
| eQTLGen | blood | CYB5B | rs72797202 | 16 | 69519790 | A | C | 0.21434 | 219.5687219 | 0.21326 | 0.0143921 | 1.12409E-49 | 0.0467 | 0.0326 | 0.1522 |
| eQTLGen | blood | CYB5B | rs77073902 | 16 | 69123280 | C | A | 0.095064 | 46.3229305 | 0.137884 | 0.0202589 | 1.00346E-11 | 0.0493 | 0.0433 | 0.2547 |
| eQTLGen | blood | CYB5B | rs78268165 | 16 | 69417302 | G | T | 0.0478191 | 17.83892335 | -0.117732 | 0.0278747 | 0.000024022 | -0.0144 | 0.0567 | 0.7999 |
| eQTLGen | blood | CYB5B | rs80095869 | 16 | 68965540 | C | T | 0.0395021 | 22.68329845 | -0.145409 | 0.0305308 | 1.90928E-06 | -0.0661 | 0.0588 | 0.2612 |
| eQTLGen | blood | CYB5B | rs8047014 | 16 | 69135049 | A | C | 0.573346 | 96.80396611 | 0.117994 | 0.0119926 | 7.64716E-23 | 0.1026 | 0.028 | 0.000249299 |
| eQTLGen | blood | CYB5B | rs8055800 | 16 | 68948094 | C | T | 0.904148 | 19.88712946 | -0.0900957 | 0.0202031 | 8.21883E-06 | 0.0209 | 0.0459 | 0.6485 |
| eQTLGen | blood | CYB5B | rs8061222 | 16 | 69435410 | G | A | 0.0428489 | 42.41280661 | -0.191109 | 0.0293449 | 7.38584E-11 | -0.0765 | 0.0773 | 0.3221 |
| eQTLGen | blood | NDUFA6 | rs1001586 | 22 | 42670293 | T | G | 0.198899 | 683.8949093 | -0.380806 | 0.0145616 | 9.5061E-151 | -0.0424 | 0.0456 | 0.3526 |
| eQTLGen | blood | NDUFA6 | rs11090046 | 22 | 41769083 | C | T | 0.0940344 | 35.73615712 | -0.121745 | 0.0203656 | 2.25621E-09 | -0.0373 | 0.0385 | 0.3321 |
| eQTLGen | blood | NDUFA6 | rs11090100 | 22 | 42811647 | C | A | 0.376297 | 114.530002 | -0.130947 | 0.0122359 | 9.96093E-27 | -0.0296 | 0.0299 | 0.3231 |
| eQTLGen | blood | NDUFA6 | rs111988926 | 22 | 42537280 | C | G | 0.0882353 | 25.95483629 | -0.106805 | 0.0209644 | 3.49478E-07 | -0.0194 | 0.0398 | 0.6261 |
| eQTLGen | blood | NDUFA6 | rs112391039 | 22 | 41610240 | T | C | 0.0153415 | 21.03697322 | 0.22194 | 0.0483887 | 4.50952E-06 | 0.1828 | 0.1454 | 0.2087 |
| eQTLGen | blood | NDUFA6 | rs112991199 | 22 | 42649298 | C | G | 0.0714286 | 17.25741801 | -0.0959451 | 0.0230959 | 3.26137E-05 | 0.1088 | 0.067 | 0.1047 |
| eQTLGen | blood | NDUFA6 | rs113354489 | 22 | 41576462 | A | C | 0.0561858 | 46.14896883 | -0.175291 | 0.0258035 | 1.09522E-11 | -0.1277 | 0.0973 | 0.1897 |
| eQTLGen | blood | NDUFA6 | rs113795690 | 22 | 41770056 | A | G | 0.0826825 | 117.8523284 | -0.233637 | 0.0215215 | 1.86337E-27 | -0.1058 | 0.0675 | 0.1169 |
| eQTLGen | blood | NDUFA6 | rs115554035 | 22 | 42149570 | A | G | 0.0202814 | 44.77758952 | -0.28209 | 0.0421558 | 2.2075E-11 | 0.1067 | 0.0969 | 0.2711 |
| eQTLGen | blood | NDUFA6 | rs116986744 | 22 | 41741098 | T | C | 0.0174633 | 685.9699941 | 1.16213 | 0.0443713 | 3.3651E-151 | 0.0606 | 0.1289 | 0.638 |
| eQTLGen | blood | NDUFA6 | rs117206004 | 22 | 42636965 | A | G | 0.0157072 | 43.8522876 | -0.316487 | 0.0477925 | 3.53916E-11 | 0.0307 | 0.1462 | 0.8339 |
| eQTLGen | blood | NDUFA6 | rs117247809 | 22 | 42796836 | C | T | 0.0395421 | 1303.428158 | 1.05497 | 0.0292211 | 1E-200 | 0.184 | 0.0903 | 0.0415901 |
| eQTLGen | blood | NDUFA6 | rs117644134 | 22 | 42384019 | T | C | 0.0165118 | 51.44608945 | -0.334387 | 0.0466201 | 7.34852E-13 | -0.1631 | 0.2558 | 0.523801 |
| eQTLGen | blood | NDUFA6 | rs13056216 | 22 | 42131447 | T | C | 0.352979 | 20.27622362 | 0.0560392 | 0.0124451 | 6.70641E-06 | 0.0259 | 0.0298 | 0.3855 |
| eQTLGen | blood | NDUFA6 | rs133291 | 22 | 42269628 | T | C | 0.243686 | 302.0653477 | -0.238414 | 0.0137177 | 1.16547E-67 | -0.0018 | 0.034 | 0.9573 |
| eQTLGen | blood | NDUFA6 | rs134897 | 22 | 42681860 | C | T | 0.722172 | 31.18122563 | -0.0741144 | 0.0132726 | 2.35321E-08 | -0.0243 | 0.0284 | 0.3935 |
| eQTLGen | blood | NDUFA6 | rs1354034 | 3 | 56849749 | C | T | 0.618137 | 44.02976135 | 0.0811607 | 0.0122313 | 3.23221E-11 | 0.0424 | 0.0308 | 0.1678 |
| eQTLGen | blood | NDUFA6 | rs139391966 | 22 | 42752423 | A | G | 0.0198351 | 26.95997019 | -0.221423 | 0.0426445 | 2.07601E-07 | 0.1315 | 0.1901 | 0.4893 |
| eQTLGen | blood | NDUFA6 | rs139503 | 22 | 41649941 | A | G | 0.464368 | 47.15806602 | -0.0818155 | 0.011914 | 6.53883E-12 | -0.0227 | 0.0281 | 0.4195 |
| eQTLGen | blood | NDUFA6 | rs139696415 | 22 | 42331208 | T | C | 0.0301871 | 86.03123754 | -0.321661 | 0.0346793 | 1.7697E-20 | 0.0128 | 0.084 | 0.8786 |
| eQTLGen | blood | NDUFA6 | rs139764778 | 22 | 41665995 | A | G | 0.03042 | 54.30212781 | 0.254887 | 0.0345891 | 1.72187E-13 | 0.0337 | 0.0754 | 0.6552 |
| eQTLGen | blood | NDUFA6 | rs140413410 | 22 | 41959827 | A | G | 0.0129282 | 42.23303759 | 0.341884 | 0.0526081 | 8.10774E-11 | 0.1824 | 0.1575 | 0.2468 |
| eQTLGen | blood | NDUFA6 | rs141621691 | 22 | 43053140 | T | C | 0.0200921 | 117.2803401 | 0.457462 | 0.0422418 | 2.4923E-27 | 0.3378 | 0.1021 | 0.000939096 |
| eQTLGen | blood | NDUFA6 | rs143174549 | 22 | 41909029 | A | C | 0.0201678 | 38.80306248 | -0.263376 | 0.0422808 | 4.6822E-10 | 0.1045 | 0.0969 | 0.2808 |
| eQTLGen | blood | NDUFA6 | rs143256776 | 22 | 42262411 | A | G | 0.024178 | 1518.459265 | 1.43471 | 0.0368182 | 1E-200 | 0.1568 | 0.1192 | 0.1882 |
| eQTLGen | blood | NDUFA6 | rs143879709 | 22 | 42632589 | T | C | 0.0243392 | 86.3000569 | -0.357704 | 0.0385051 | 1.54277E-20 | 0.0127 | 0.0915 | 0.8899 |
| eQTLGen | blood | NDUFA6 | rs146140905 | 22 | 42134539 | T | C | 0.114716 | 330.4370664 | 0.33559 | 0.0184614 | 7.73749E-74 | 0.0408 | 0.0486 | 0.4016 |
| eQTLGen | blood | NDUFA6 | rs146741716 | 22 | 42082501 | T | G | 0.0145553 | 25.86536427 | 0.252511 | 0.0496502 | 3.66471E-07 | 0.143 | 0.1182 | 0.2263 |
| eQTLGen | blood | NDUFA6 | rs146824396 | 22 | 41922643 | C | T | 0.0185487 | 742.8977225 | 1.17187 | 0.0429947 | 1.4028E-163 | 0.0927 | 0.129 | 0.4725 |
| eQTLGen | blood | NDUFA6 | rs147368801 | 22 | 42199272 | T | A | 0.01667 | 31.66435743 | 0.261292 | 0.0464345 | 1.83371E-08 | 0.1357 | 0.1193 | 0.2554 |
| eQTLGen | blood | NDUFA6 | rs149013899 | 22 | 42542651 | T | C | 0.0343172 | 2035.025268 | 1.37883 | 0.0305651 | 1E-200 | 0.1686 | 0.0993 | 0.0896004 |
| eQTLGen | blood | NDUFA6 | rs149228586 | 22 | 41608058 | T | C | 0.0200956 | 17.13380592 | -0.175455 | 0.0423876 | 3.48217E-05 | 0.173 | 0.091 | 0.0572097 |
| eQTLGen | blood | NDUFA6 | rs149567399 | 22 | 42311204 | T | G | 0.0389519 | 40.97133522 | 0.196616 | 0.030717 | 1.54561E-10 | -0.0306 | 0.0537 | 0.5695 |
| eQTLGen | blood | NDUFA6 | rs1548304 | 22 | 42691238 | T | C | 0.474648 | 41.19824486 | -0.0763908 | 0.0119015 | 1.37531E-10 | -0.042 | 0.0284 | 0.1396 |
| eQTLGen | blood | NDUFA6 | rs17002308 | 22 | 41536452 | C | T | 0.0222662 | 19.05856759 | -0.17598 | 0.0403105 | 1.26771E-05 | 0.0144 | 0.0983 | 0.8837 |
| eQTLGen | blood | NDUFA6 | rs17433014 | 22 | 41499822 | A | G | 0.0387509 | 23.69922423 | -0.149999 | 0.0308121 | 1.1251E-06 | 0.0796 | 0.0522 | 0.1278 |
| eQTLGen | blood | NDUFA6 | rs183748472 | 22 | 42041925 | A | G | 0.0187846 | 33.47892205 | -0.253357 | 0.0437872 | 7.19963E-09 | 0.1294 | 0.1057 | 0.221 |
| eQTLGen | blood | NDUFA6 | rs184348183 | 22 | 42181368 | A | G | 0.0212705 | 76.09958646 | -0.358879 | 0.0411393 | 2.69464E-18 | -0.157 | 0.1507 | 0.2978 |
| eQTLGen | blood | NDUFA6 | rs186533270 | 22 | 41657075 | C | T | 0.0211869 | 22.49323566 | -0.195858 | 0.0412967 | 2.1078E-06 | 0.0729 | 0.0986 | 0.459501 |
| eQTLGen | blood | NDUFA6 | rs2267436 | 22 | 41905278 | C | T | 0.179429 | 176.9011995 | 0.205022 | 0.0154147 | 2.30834E-40 | 0.0784 | 0.0407 | 0.0536698 |
| eQTLGen | blood | NDUFA6 | rs41311445 | 22 | 42070374 | C | A | 0.105052 | 70.41704871 | -0.162481 | 0.0193626 | 4.79623E-17 | 0.0134 | 0.0411 | 0.744099 |
| eQTLGen | blood | NDUFA6 | rs4822099 | 22 | 42659964 | A | G | 0.0405695 | 2341.8732 | 1.35203 | 0.0279386 | 1E-200 | 0.188 | 0.0961 | 0.0505499 |
| eQTLGen | blood | NDUFA6 | rs4822112 | 22 | 42731540 | T | G | 0.842941 | 92.71384236 | 0.156988 | 0.016304 | 6.03115E-22 | -0.0441 | 0.0323 | 0.1721 |
| eQTLGen | blood | NDUFA6 | rs55758879 | 22 | 42392091 | A | G | 0.0386495 | 1950.756759 | 1.27827 | 0.0289415 | 1E-200 | 0.1498 | 0.098 | 0.1263 |
| eQTLGen | blood | NDUFA6 | rs5751086 | 22 | 41768862 | T | C | 0.254738 | 26.40831708 | -0.0701306 | 0.013647 | 2.76198E-07 | -0.026 | 0.0314 | 0.4085 |
| eQTLGen | blood | NDUFA6 | rs5751221 | 22 | 42516366 | T | C | 0.204563 | 781.9363836 | -0.401612 | 0.0143622 | 4.529E-172 | -0.0558 | 0.043 | 0.1951 |
| eQTLGen | blood | NDUFA6 | rs5751233 | 22 | 42566101 | T | G | 0.0277722 | 84.11829331 | -0.331215 | 0.0361131 | 4.66015E-20 | 0.0798 | 0.1375 | 0.5618 |
| eQTLGen | blood | NDUFA6 | rs5751273 | 22 | 42850301 | C | T | 0.279911 | 231.8374637 | 0.200214 | 0.0131493 | 2.36974E-52 | 0.0239 | 0.0323 | 0.4601 |
| eQTLGen | blood | NDUFA6 | rs5758563 | 22 | 42448761 | T | G | 0.0245505 | 34.8737648 | -0.226843 | 0.0384128 | 3.51366E-09 | -0.2179 | 0.1285 | 0.0899001 |
| eQTLGen | blood | NDUFA6 | rs5758701 | 22 | 42690262 | G | A | 0.0250305 | 24.67896343 | -0.189103 | 0.0380658 | 6.76472E-07 | 0.0673 | 0.213 | 0.7519 |
| eQTLGen | blood | NDUFA6 | rs5758746 | 22 | 42873942 | G | A | 0.835832 | 72.22396078 | -0.136199 | 0.0160263 | 1.92221E-17 | -0.0147 | 0.034 | 0.665001 |
| eQTLGen | blood | NDUFA6 | rs5758871 | 22 | 43095600 | C | T | 0.438793 | 17.9301138 | 0.0507539 | 0.0119861 | 2.29161E-05 | 0.0032 | 0.0281 | 0.9082 |
| eQTLGen | blood | NDUFA6 | rs5759137 | 22 | 43472319 | A | G | 0.223849 | 23.3307329 | -0.0689128 | 0.0142671 | 1.36261E-06 | 0.0923 | 0.0351 | 0.00856308 |
| eQTLGen | blood | NDUFA6 | rs5996171 | 22 | 42872086 | T | C | 0.832388 | 46.48362909 | -0.108459 | 0.015908 | 9.24485E-12 | 0.0233 | 0.0394 | 0.5536 |
| eQTLGen | blood | NDUFA6 | rs6002295 | 22 | 41640850 | C | T | 0.554523 | 81.48084215 | 0.107783 | 0.0119405 | 1.76767E-19 | 0.0268 | 0.0288 | 0.3512 |
| eQTLGen | blood | NDUFA6 | rs6002546 | 22 | 42333408 | G | T | 0.213266 | 359.7230236 | 0.272137 | 0.0143484 | 3.24564E-80 | 0.0282 | 0.0342 | 0.41 |
| eQTLGen | blood | NDUFA6 | rs6002745 | 22 | 42871115 | G | T | 0.486591 | 43.50040141 | 0.078417 | 0.0118895 | 4.24131E-11 | 0.0133 | 0.0283 | 0.6389 |
| eQTLGen | blood | NDUFA6 | rs6002850 | 22 | 43041545 | G | A | 0.268556 | 20.06251123 | 0.0601062 | 0.0134192 | 7.49946E-06 | 0.0225 | 0.0306 | 0.4619 |
| eQTLGen | blood | NDUFA6 | rs60530919 | 22 | 42051845 | G | A | 0.0268255 | 92.82914727 | 0.353748 | 0.0367157 | 5.71084E-22 | -0.1553 | 0.1022 | 0.1287 |
| eQTLGen | blood | NDUFA6 | rs61342075 | 22 | 41496846 | T | C | 0.371192 | 130.0116923 | 0.139826 | 0.012263 | 4.07662E-30 | -0.0047 | 0.0306 | 0.8768 |
| eQTLGen | blood | NDUFA6 | rs62236533 | 22 | 41992169 | A | G | 0.0861907 | 191.1038622 | -0.291206 | 0.0210652 | 1.82642E-43 | -0.1012 | 0.0632 | 0.1094 |
| eQTLGen | blood | NDUFA6 | rs62238186 | 22 | 41778714 | T | C | 0.0165578 | 16.86913853 | -0.19145 | 0.0466132 | 4.00479E-05 | 0.0137 | 0.1242 | 0.9123 |
| eQTLGen | blood | NDUFA6 | rs62238599 | 22 | 42504168 | G | A | 0.0210794 | 39.62079407 | -0.260432 | 0.0413745 | 3.08191E-10 | -0.1106 | 0.1608 | 0.4916 |
| eQTLGen | blood | NDUFA6 | rs62238724 | 22 | 41569004 | G | A | 0.0273829 | 21.66543713 | -0.169623 | 0.0364419 | 3.24609E-06 | -0.2359 | 0.127 | 0.0631902 |
| eQTLGen | blood | NDUFA6 | rs62239665 | 22 | 42855719 | G | A | 0.0162738 | 18.44614863 | -0.201898 | 0.0470088 | 1.74691E-05 | -0.1672 | 0.1827 | 0.3601 |
| eQTLGen | blood | NDUFA6 | rs62240986 | 22 | 42294253 | T | G | 0.0159115 | 67.66214143 | -0.390307 | 0.0474497 | 1.9391E-16 | 0.0108 | 0.1232 | 0.9299 |
| eQTLGen | blood | NDUFA6 | rs7286414 | 22 | 42716632 | A | G | 0.284703 | 101.7317416 | 0.132548 | 0.0131415 | 6.3577E-24 | 0.0007 | 0.0329 | 0.984 |
| eQTLGen | blood | NDUFA6 | rs7292894 | 22 | 42214719 | T | C | 0.0213419 | 16.58522127 | -0.167617 | 0.0411583 | 4.64911E-05 | -0.1522 | 0.0906 | 0.092871 |
| eQTLGen | blood | NDUFA6 | rs73173008 | 22 | 42798571 | A | G | 0.0231831 | 49.8874394 | -0.278857 | 0.0394808 | 1.62705E-12 | -0.095 | 0.1862 | 0.609799 |
| eQTLGen | blood | NDUFA6 | rs73435512 | 22 | 42758726 | T | C | 0.0184089 | 21.67552509 | 0.205976 | 0.0442417 | 3.23043E-06 | 0.0962 | 0.1729 | 0.578 |
| eQTLGen | blood | NDUFA6 | rs738140 | 22 | 41884954 | G | A | 0.318021 | 136.5817822 | -0.14864 | 0.0127186 | 1.48525E-31 | -0.0126 | 0.03 | 0.675499 |
| eQTLGen | blood | NDUFA6 | rs74322737 | 22 | 41697981 | G | T | 0.0284557 | 63.08653459 | 0.28368 | 0.0357158 | 1.9797E-15 | -0.0618 | 0.0766 | 0.4198 |
| eQTLGen | blood | NDUFA6 | rs76277210 | 22 | 42447531 | A | G | 0.025635 | 98.25752088 | -0.372001 | 0.0375285 | 3.66944E-23 | 0.0144 | 0.0906 | 0.8741 |
| eQTLGen | blood | NDUFA6 | rs8073060 | 17 | 33875262 | A | T | 0.305723 | 25.45217314 | -0.0651155 | 0.0129069 | 4.53524E-07 | 0.0386 | 0.0327 | 0.2378 |
| eQTLGen | blood | NDUFA6 | rs8138990 | 22 | 41597377 | C | A | 0.335119 | 127.1897491 | 0.141564 | 0.0125524 | 1.68927E-29 | -0.006 | 0.0307 | 0.8464 |
| eQTLGen | blood | NDUFA6 | rs9611560 | 22 | 41750622 | C | T | 0.76771 | 138.5755281 | -0.165102 | 0.0140252 | 5.45507E-32 | -0.0384 | 0.0383 | 0.316 |
| eQTLGen | blood | NDUFA6 | rs9611776 | 22 | 42757523 | T | C | 0.0259844 | 29.43719966 | -0.202769 | 0.0373726 | 5.76966E-08 | -0.0887 | 0.0707 | 0.2092 |
| eQTLGen | blood | NDUFA6 | rs9620006 | 22 | 42399547 | A | T | 0.27493 | 520.9385188 | -0.29879 | 0.013091 | 2.6363E-115 | -0.0181 | 0.0407 | 0.6561 |
| eQTLGen | blood | NDUFAF3 | rs113592495 | 3 | 49146656 | G | T | 0.135969 | 34.84082848 | 0.102369 | 0.017343 | 3.58253E-09 | -0.0058 | 0.0391 | 0.8823 |
| eQTLGen | blood | NDUFAF3 | rs114182490 | 3 | 48865926 | A | G | 0.03977 | 16.46090019 | 0.123496 | 0.0304387 | 4.97073E-05 | -0.0865 | 0.0659 | 0.1895 |
| eQTLGen | blood | NDUFAF3 | rs11553699 | 12 | 122216910 | G | A | 0.10963 | 28.95509286 | 0.102403 | 0.0190305 | 7.4073E-08 | -0.0857 | 0.0459 | 0.0618401 |
| eQTLGen | blood | NDUFAF3 | rs115770773 | 3 | 49891362 | C | T | 0.0995551 | 53.15854195 | -0.144662 | 0.0198412 | 3.07185E-13 | 0.0843 | 0.0564 | 0.1351 |
| eQTLGen | blood | NDUFAF3 | rs115869055 | 3 | 48456870 | G | T | 0.0423609 | 19.5107486 | 0.130436 | 0.0295298 | 1.00081E-05 | 0.022 | 0.0701 | 0.7533 |
| eQTLGen | blood | NDUFAF3 | rs11602954 | 11 | 202856 | A | G | 0.211758 | 28.96162844 | 0.0783179 | 0.0145529 | 7.39095E-08 | -0.0107 | 0.0311 | 0.730099 |
| eQTLGen | blood | NDUFAF3 | rs116786562 | 3 | 48141643 | T | C | 0.0783847 | 34.57345141 | -0.130045 | 0.0221168 | 4.1027E-09 | 0.0746 | 0.048 | 0.1198 |
| eQTLGen | blood | NDUFAF3 | rs11716948 | 3 | 49431903 | T | C | 0.247238 | 20.91065672 | 0.0630404 | 0.0137859 | 4.81715E-06 | 0.0315 | 0.0349 | 0.3669 |
| eQTLGen | blood | NDUFAF3 | rs1264191 | 3 | 48649457 | C | T | 0.812455 | 109.1806635 | 0.158705 | 0.0151886 | 1.48081E-25 | -0.0789 | 0.0351 | 0.0247298 |
| eQTLGen | blood | NDUFAF3 | rs12715430 | 3 | 48942855 | C | A | 0.754605 | 182.8404922 | 0.18582 | 0.0137422 | 1.16305E-41 | -0.0836 | 0.0338 | 0.0134899 |
| eQTLGen | blood | NDUFAF3 | rs13062429 | 3 | 49559485 | G | A | 0.68947 | 27.20569288 | -0.0670265 | 0.0128504 | 1.83029E-07 | 0.0255 | 0.0283 | 0.3677 |
| eQTLGen | blood | NDUFAF3 | rs13316065 | 3 | 49884913 | T | C | 0.289267 | 40.97267667 | 0.0838997 | 0.0131073 | 1.54358E-10 | -0.0142 | 0.0324 | 0.662 |
| eQTLGen | blood | NDUFAF3 | rs1354034 | 3 | 56849749 | C | T | 0.618137 | 293.3486077 | 0.20767 | 0.012125 | 9.23847E-66 | 0.0424 | 0.0308 | 0.1678 |
| eQTLGen | blood | NDUFAF3 | rs139125439 | 3 | 49995603 | G | A | 0.118754 | 61.61454678 | -0.144101 | 0.018358 | 4.1735E-15 | 0.0964 | 0.0418 | 0.0210601 |
| eQTLGen | blood | NDUFAF3 | rs140746667 | 3 | 49734204 | T | C | 0.0576521 | 18.40825303 | 0.109485 | 0.0255181 | 1.78349E-05 | -0.068 | 0.0506 | 0.1786 |
| eQTLGen | blood | NDUFAF3 | rs140796186 | 3 | 49272407 | A | G | 0.0172275 | 22.05528719 | -0.214647 | 0.0457055 | 2.64789E-06 | -0.2277 | 0.2337 | 0.3298 |
| eQTLGen | blood | NDUFAF3 | rs144790640 | 3 | 48977464 | A | C | 0.0405012 | 29.57178356 | -0.164011 | 0.0301602 | 5.3822E-08 | 0.0865 | 0.0646 | 0.1806 |
| eQTLGen | blood | NDUFAF3 | rs17825630 | 3 | 56950055 | A | G | 0.108121 | 27.14407932 | 0.09976 | 0.0191478 | 1.88838E-07 | 0.0091 | 0.0486 | 0.8515 |
| eQTLGen | blood | NDUFAF3 | rs182210511 | 3 | 48962629 | T | C | 0.0261463 | 29.46008063 | -0.202235 | 0.0372597 | 5.70532E-08 | -0.0453 | 0.1192 | 0.7038 |
| eQTLGen | blood | NDUFAF3 | rs2109181 | 3 | 48580173 | G | A | 0.17454 | 31.55296688 | 0.08798 | 0.0156626 | 1.9408E-08 | -0.0163 | 0.0365 | 0.656 |
| eQTLGen | blood | NDUFAF3 | rs2230593 | 3 | 49940078 | T | C | 0.0687182 | 34.20865475 | -0.137439 | 0.0234986 | 4.95131E-09 | 0.1328 | 0.0708 | 0.0608793 |
| eQTLGen | blood | NDUFAF3 | rs3135952 | 3 | 48510210 | T | C | 0.0305846 | 23.56435187 | -0.16765 | 0.0345363 | 1.20751E-06 | 0.1655 | 0.0788 | 0.0357602 |
| eQTLGen | blood | NDUFAF3 | rs342296 | 7 | 106372903 | A | G | 0.441001 | 28.53501532 | -0.0639688 | 0.0119751 | 9.19263E-08 | 0.0162 | 0.0281 | 0.5632 |
| eQTLGen | blood | NDUFAF3 | rs35762866 | 3 | 49690627 | A | G | 0.118472 | 56.57568415 | -0.13825 | 0.0183802 | 5.40256E-14 | 0.0901 | 0.043 | 0.0362602 |
| eQTLGen | blood | NDUFAF3 | rs3731529 | 3 | 48268509 | T | A | 0.234763 | 99.61303302 | -0.139657 | 0.0139928 | 1.84969E-23 | 0.0717 | 0.0325 | 0.0272898 |
| eQTLGen | blood | NDUFAF3 | rs3733606 | 4 | 6883012 | T | C | 0.167763 | 27.01821518 | -0.0827153 | 0.0159132 | 2.01442E-07 | -0.048 | 0.0436 | 0.2709 |
| eQTLGen | blood | NDUFAF3 | rs3749237 | 3 | 49770032 | A | G | 0.30097 | 43.73819511 | 0.085683 | 0.0129558 | 3.75578E-11 | -0.0146 | 0.0323 | 0.6519 |
| eQTLGen | blood | NDUFAF3 | rs4688688 | 3 | 50042690 | G | C | 0.828967 | 21.98772815 | -0.0740607 | 0.0157942 | 2.74543E-06 | -0.0263 | 0.0356 | 0.4607 |
| eQTLGen | blood | NDUFAF3 | rs55935606 | 3 | 49565505 | G | A | 0.190553 | 140.2942364 | -0.178614 | 0.0150798 | 2.29509E-32 | 0.1085 | 0.0379 | 0.00422299 |
| eQTLGen | blood | NDUFAF3 | rs56176327 | 3 | 49706736 | C | G | 0.0965812 | 53.3776607 | -0.146931 | 0.020111 | 2.75233E-13 | 0.0748 | 0.0558 | 0.18 |
| eQTLGen | blood | NDUFAF3 | rs62263033 | 3 | 48370424 | C | T | 0.0348859 | 24.66617272 | -0.160954 | 0.0324079 | 6.81365E-07 | 0.1287 | 0.0751 | 0.0866902 |
| eQTLGen | blood | NDUFAF3 | rs6442112 | 3 | 48316034 | G | A | 0.699753 | 65.43870435 | 0.104796 | 0.0129547 | 5.98549E-16 | -0.0477 | 0.0309 | 0.1228 |
| eQTLGen | blood | NDUFAF3 | rs6584485 | 10 | 103937244 | T | C | 0.838854 | 19.0445888 | -0.0705966 | 0.016177 | 1.27711E-05 | 0.0558 | 0.0544 | 0.3051 |
| eQTLGen | blood | NDUFAF3 | rs6803264 | 3 | 49254427 | C | T | 0.771596 | 141.4799362 | 0.167795 | 0.0141069 | 1.2627E-32 | -0.0992 | 0.0357 | 0.00550098 |
| eQTLGen | blood | NDUFAF3 | rs71324982 | 3 | 49669948 | A | G | 0.232896 | 26.35575764 | 0.0722221 | 0.014068 | 2.8425E-07 | 0.0315 | 0.0362 | 0.3847 |
| eQTLGen | blood | NDUFAF3 | rs730566 | 3 | 48487048 | A | C | 0.293698 | 82.13786133 | -0.118089 | 0.0130298 | 1.26678E-19 | 0.0612 | 0.0313 | 0.0509601 |
| eQTLGen | blood | NDUFAF3 | rs73082337 | 3 | 49009570 | G | C | 0.127844 | 16.61783376 | 0.0726171 | 0.0178136 | 4.57383E-05 | -0.0569 | 0.0383 | 0.1375 |
| eQTLGen | blood | NDUFAF3 | rs7429596 | 3 | 48808531 | C | T | 0.754964 | 163.4373729 | 0.175891 | 0.0137584 | 2.00401E-37 | -0.085 | 0.0338 | 0.01199 |
| eQTLGen | blood | NDUFAF3 | rs7621347 | 3 | 49110889 | C | T | 0.770288 | 173.1367927 | 0.185043 | 0.014063 | 1.52686E-39 | -0.0903 | 0.0339 | 0.00762202 |
| eQTLGen | blood | NDUFAF3 | rs7623687 | 3 | 49448566 | C | A | 0.146398 | 21.30657845 | 0.0776566 | 0.0168237 | 3.9158E-06 | -0.0311 | 0.0333 | 0.3499 |
| eQTLGen | blood | NDUFAF3 | rs76772800 | 3 | 48250911 | A | G | 0.0477102 | 17.70990607 | 0.117433 | 0.027905 | 2.57413E-05 | -0.0232 | 0.0701 | 0.7406 |
| eQTLGen | blood | NDUFAF3 | rs773125 | 12 | 56401085 | G | A | 0.370039 | 22.36352961 | -0.0582491 | 0.0123174 | 2.25408E-06 | -0.0155 | 0.0284 | 0.5857 |
| eQTLGen | blood | NDUFAF3 | rs77860278 | 3 | 49089437 | G | A | 0.0161038 | 28.35244547 | -0.251516 | 0.0472357 | 1.0106E-07 | -0.1623 | 0.2258 | 0.4722 |
| eQTLGen | blood | NDUFAF3 | rs7803454 | 7 | 99991548 | T | C | 0.189533 | 19.38719477 | 0.0668182 | 0.0151753 | 0.000010676 | 0.0354 | 0.0335 | 0.2905 |
| eQTLGen | blood | NDUFAF3 | rs7961894 | 12 | 122365583 | T | C | 0.105151 | 27.99790289 | 0.102564 | 0.0193835 | 1.21521E-07 | -0.0951 | 0.0476 | 0.0455397 |
| eQTLGen | blood | NDUFAF3 | rs9399137 | 6 | 135419018 | C | T | 0.264393 | 33.79682141 | 0.0783637 | 0.0134796 | 6.12167E-09 | 0.0249 | 0.0296 | 0.4002 |
| eQTLGen | blood | NDUFAF3 | rs9854297 | 3 | 49417896 | T | A | 0.769447 | 145.1964317 | 0.169405 | 0.0140588 | 1.93955E-33 | -0.1066 | 0.0359 | 0.00295502 |
| GTEx | blood | CYB5B | rs246147 | 16 | 69468842 | C | T | 0.4496 | 32.66470215 | -0.125859 | 0.0220214 | 1.91E-08 | -0.0717 | 0.0282 | 0.0109199 |
| GTEx | blood | NDUFA6 | rs116886599 | 22 | 42357105 | C | T | 0.0186 | 148.6499889 | 0.694361 | 0.0569512 | 4.93E-30 | 0.1854 | 0.1029 | 0.0716193 |
| GTEx | blood | NDUFA6 | rs117518412 | 22 | 42637023 | A | G | 0.02154 | 255.1839003 | 0.776334 | 0.0485984 | 1.64E-46 | 0.1652 | 0.0948 | 0.0814198 |
| GTEx | blood | NDUFA6 | rs117716410 | 22 | 41703510 | A | C | 0.01207 | 23.98610065 | 0.424882 | 0.0867538 | 1.32E-06 | 0.0523 | 0.1284 | 0.684 |
| GTEx | blood | NDUFA6 | rs117943151 | 22 | 42759320 | A | G | 0.02496 | 105.5438238 | 0.544832 | 0.053033 | 1.52E-22 | 0.1727 | 0.0891 | 0.0526102 |
| GTEx | blood | NDUFA6 | rs139521 | 22 | 41661977 | C | T | 0.8332 | 17.05233123 | -0.108858 | 0.0263614 | 4.28E-05 | -0.0225 | 0.0376 | 0.5495 |
| GTEx | blood | NDUFA6 | rs146824396 | 22 | 41922643 | C | T | 0.01194 | 45.51663722 | 0.541966 | 0.0803317 | 4.30E-11 | 0.0927 | 0.129 | 0.4725 |
| GTEx | blood | NDUFA6 | rs183413369 | 22 | 41808162 | A | G | 0.0119 | 33.5002128 | 0.49554 | 0.085616 | 1.28E-08 | 0.0776 | 0.1293 | 0.5486 |
| GTEx | blood | NDUFA6 | rs1989202 | 22 | 42486080 | A | G | 0.02067 | 252.3829349 | 0.747752 | 0.0470682 | 4.14E-46 | 0.1494 | 0.097 | 0.1237 |
| GTEx | blood | NDUFA6 | rs6002708 | 22 | 42768154 | C | T | 0.4719 | 17.8460498 | -0.0887639 | 0.0210119 | 2.86E-05 | -0.0495 | 0.0281 | 0.0785001 |
| GTEx | blood | NDUFA6 | rs73163373 | 22 | 42232588 | A | G | 0.1139 | 22.41700384 | 0.145364 | 0.0307021 | 2.88E-06 | 0.0272 | 0.044 | 0.5362 |
| GTEx | blood | NDUFA6 | rs73419054 | 22 | 42866999 | G | T | 0.02968 | 36.77255573 | 0.292303 | 0.0482027 | 2.66E-09 | 0.2284 | 0.0821 | 0.00538295 |
| GTEx | blood | NDUFA6 | rs9607871 | 22 | 42436322 | A | G | 0.1101 | 23.74079952 | -0.131718 | 0.0270332 | 1.49E-06 | -0.0355 | 0.0449 | 0.4294 |
| GTEx | aorta | NDUFA6 | rs116886599 | 22 | 42357105 | C | T | 0.0186 | 162.5151669 | 1.32712 | 0.104103 | 1.54E-29 | 0.1854 | 0.1029 | 0.0716193 |
| GTEx | aorta | NDUFA6 | rs117518412 | 22 | 42637023 | A | G | 0.02154 | 416.0565908 | 1.56697 | 0.0768218 | 6.86E-57 | 0.1652 | 0.0948 | 0.0814198 |
| GTEx | aorta | NDUFA6 | rs117716410 | 22 | 41703510 | A | C | 0.01207 | 31.02170731 | 0.980225 | 0.175992 | 6.09E-08 | 0.0523 | 0.1284 | 0.684 |
| GTEx | aorta | NDUFA6 | rs117943151 | 22 | 42759320 | A | G | 0.02496 | 77.34206359 | 1.05666 | 0.120151 | 1.63E-16 | 0.1727 | 0.0891 | 0.0526102 |
| GTEx | aorta | NDUFA6 | rs139043947 | 22 | 42480421 | A | G | 0.02065 | 508.0320452 | 1.59934 | 0.070957 | 2.37E-64 | 0.1497 | 0.0971 | 0.1231 |
| GTEx | aorta | NDUFA6 | rs146140905 | 22 | 42134539 | T | C | 0.09092 | 25.94466255 | 0.352724 | 0.0692486 | 6.55E-07 | 0.0408 | 0.0486 | 0.4016 |
| GTEx | aorta | NDUFA6 | rs192756643 | 22 | 41922643 | T | C | 0.01194 | 51.28199662 | 1.09307 | 0.152639 | 7.38E-12 | 0.0927 | 0.129 | 0.4725 |
| GTEx | aorta | NDUFA6 | rs2269657 | 22 | 42264269 | T | G | 0.2201 | 38.75391424 | 0.309743 | 0.0497558 | 1.80E-09 | 0.0259 | 0.0337 | 0.4422 |
| GTEx | aorta | NDUFA6 | rs73419054 | 22 | 42866999 | G | T | 0.02968 | 44.73237067 | 0.710818 | 0.106279 | 1.27E-10 | 0.2284 | 0.0821 | 0.00538295 |
| GTEx | tibial artery | NDUFA6 | rs1001586 | 22 | 42670293 | T | G | 0.1066 | 26.23656232 | -0.20566 | 0.040151 | 4.68E-07 | -0.0424 | 0.0456 | 0.3526 |
| GTEx | tibial artery | NDUFA6 | rs116886599 | 22 | 42357105 | C | T | 0.0186 | 402.0200326 | 1.40329 | 0.069988 | 1.18E-62 | 0.1854 | 0.1029 | 0.0716193 |
| GTEx | tibial artery | NDUFA6 | rs117518412 | 22 | 42637023 | A | G | 0.02154 | 573.4690961 | 1.41927 | 0.0592666 | 1.18E-79 | 0.1652 | 0.0948 | 0.0814198 |
| GTEx | tibial artery | NDUFA6 | rs117716410 | 22 | 41703510 | A | C | 0.01207 | 33.73696696 | 0.852492 | 0.14677 | 1.28E-08 | 0.0523 | 0.1284 | 0.684 |
| GTEx | tibial artery | NDUFA6 | rs117943151 | 22 | 42759320 | A | G | 0.02496 | 139.0438316 | 0.89445 | 0.0758543 | 8.44E-28 | 0.1727 | 0.0891 | 0.0526102 |
| GTEx | tibial artery | NDUFA6 | rs192756643 | 22 | 41922643 | T | C | 0.01194 | 70.37933384 | 1.07243 | 0.127834 | 8.18E-16 | 0.0927 | 0.129 | 0.4725 |
| GTEx | tibial artery | NDUFA6 | rs1989202 | 22 | 42486080 | A | G | 0.02067 | 641.2115001 | 1.41406 | 0.0558428 | 1.47E-85 | 0.1494 | 0.097 | 0.1237 |
| GTEx | tibial artery | NDUFA6 | rs2267440 | 22 | 42248860 | A | G | 0.7842 | 17.45180686 | 0.161712 | 0.0387099 | 3.61E-05 | 0.0017 | 0.0341 | 0.9591 |
| GTEx | tibial artery | NDUFA6 | rs2743455 | 22 | 42536064 | C | T | 0.1649 | 40.1419202 | -0.227447 | 0.0358989 | 6.28E-10 | -0.0139 | 0.0379 | 0.713099 |
| GTEx | tibial artery | NDUFA6 | rs6002546 | 22 | 42333408 | G | T | 0.2127 | 78.93032483 | 0.3046 | 0.0342853 | 2.11E-17 | 0.0282 | 0.0342 | 0.41 |
| GTEx | tibial artery | NDUFA6 | rs6002745 | 22 | 42871115 | G | T | 0.5373 | 17.70043601 | 0.13839 | 0.0328937 | 3.18E-05 | 0.0133 | 0.0283 | 0.6389 |
| GTEx | tibial artery | NDUFA6 | rs73163373 | 22 | 42232588 | A | G | 0.1139 | 41.23796835 | 0.295972 | 0.0460895 | 3.77E-10 | 0.0272 | 0.044 | 0.5362 |
| GTEx | tibial artery | NDUFA6 | rs73419054 | 22 | 42866999 | G | T | 0.02968 | 69.81079947 | 0.599333 | 0.071731 | 1.05E-15 | 0.2284 | 0.0821 | 0.00538295 |
| GTEx | tibial artery | NDUFA6 | rs9623482 | 22 | 42360574 | C | T | 0.1918 | 17.51907833 | -0.159137 | 0.0380203 | 3.49E-05 | -0.0183 | 0.0356 | 0.608199 |
| GTEx | coronary artery | NDUFA6 | rs116886599 | 22 | 42357105 | C | T | 0.0186 | 59.08549826 | 0.943459 | 0.122739 | 2.79E-12 | 0.1854 | 0.1029 | 0.0716193 |
| GTEx | coronary artery | NDUFA6 | rs117518412 | 22 | 42637023 | A | G | 0.02154 | 52.40826117 | 1.03218 | 0.142579 | 3.08E-11 | 0.1652 | 0.0948 | 0.0814198 |
| GTEx | coronary artery | NDUFA6 | rs139043947 | 22 | 42480421 | A | G | 0.02065 | 74.0739927 | 1.08607 | 0.12619 | 1.71E-14 | 0.1497 | 0.0971 | 0.1231 |
| GTEx | coronary artery | NDUFA6 | rs73419054 | 22 | 42866999 | G | T | 0.02968 | 19.65326012 | 0.51997 | 0.11729 | 1.91E-05 | 0.2284 | 0.0821 | 0.00538295 |
| GTEx | visceral omental adipose | NDUFA6 | rs117518412 | 22 | 42637023 | A | G | 0.02154 | 349.8735556 | 0.973351 | 0.0520372 | 2.05E-53 | 0.1652 | 0.0948 | 0.0814198 |
| GTEx | visceral omental adipose | NDUFA6 | rs117716410 | 22 | 41703510 | A | C | 0.01207 | 54.42682582 | 0.7471 | 0.101268 | 1.37E-12 | 0.0523 | 0.1284 | 0.684 |
| GTEx | visceral omental adipose | NDUFA6 | rs117943151 | 22 | 42759320 | A | G | 0.02496 | 91.964693 | 0.645513 | 0.0673123 | 2.49E-19 | 0.1727 | 0.0891 | 0.0526102 |
| GTEx | visceral omental adipose | NDUFA6 | rs146140905 | 22 | 42134539 | T | C | 0.09092 | 27.94416471 | 0.230805 | 0.0436616 | 2.30E-07 | 0.0408 | 0.0486 | 0.4016 |
| GTEx | visceral omental adipose | NDUFA6 | rs146824396 | 22 | 41922643 | C | T | 0.01194 | 66.54084631 | 0.78665 | 0.0964356 | 7.70E-15 | 0.0927 | 0.129 | 0.4725 |
| GTEx | visceral omental adipose | NDUFA6 | rs183413369 | 22 | 41808162 | A | G | 0.0119 | 65.45689092 | 0.806505 | 0.0996849 | 1.22E-14 | 0.0776 | 0.1293 | 0.5486 |
| GTEx | visceral omental adipose | NDUFA6 | rs1989202 | 22 | 42486080 | A | G | 0.02067 | 411.6738553 | 0.954379 | 0.0470375 | 1.36E-59 | 0.1494 | 0.097 | 0.1237 |
| GTEx | visceral omental adipose | NDUFA6 | rs2269657 | 22 | 42264269 | T | G | 0.2201 | 54.38366427 | 0.228926 | 0.0310428 | 1.40E-12 | 0.0259 | 0.0337 | 0.4422 |
| GTEx | visceral omental adipose | NDUFA6 | rs5758686 | 22 | 42655377 | T | G | 0.1066 | 29.17106413 | -0.186755 | 0.0345777 | 1.29E-07 | -0.0426 | 0.0456 | 0.3508 |
| GTEx | visceral omental adipose | NDUFA6 | rs73419054 | 22 | 42866999 | G | T | 0.02968 | 54.57651014 | 0.467605 | 0.063296 | 1.29E-12 | 0.2284 | 0.0821 | 0.00538295 |
| GTEx | visceral omental adipose | NDUFA6 | rs742086 | 22 | 42552253 | G | T | 0.1702 | 37.76135681 | -0.191079 | 0.0310949 | 2.35E-09 | -0.0209 | 0.0376 | 0.579301 |
| GTEx | visceral omental adipose | NDUFA6 | rs8140914 | 22 | 42314392 | T | C | 0.01865 | 182.9660165 | 0.912458 | 0.0674571 | 2.43E-33 | 0.1803 | 0.1028 | 0.0795298 |
| GTEx | visceral omental adipose | NDUFA6 | rs9607871 | 22 | 42436322 | A | G | 0.1101 | 26.76123643 | -0.184498 | 0.0356647 | 4.05E-07 | -0.0355 | 0.0449 | 0.4294 |
| GTEx | tibial nerve | NDUFA6 | rs116886599 | 22 | 42357105 | C | T | 0.0186 | 271.7111789 | 1.31387 | 0.0797074 | 4.15E-46 | 0.1854 | 0.1029 | 0.0716193 |
| GTEx | tibial nerve | NDUFA6 | rs117518412 | 22 | 42637023 | A | G | 0.02154 | 544.1588173 | 1.37823 | 0.0590825 | 1.56E-74 | 0.1652 | 0.0948 | 0.0814198 |
| GTEx | tibial nerve | NDUFA6 | rs117716410 | 22 | 41703510 | A | C | 0.01207 | 25.98623019 | 0.694588 | 0.136256 | 5.51E-07 | 0.0523 | 0.1284 | 0.684 |
| GTEx | tibial nerve | NDUFA6 | rs117943151 | 22 | 42759320 | A | G | 0.02496 | 99.59297364 | 0.813671 | 0.0815332 | 6.47E-21 | 0.1727 | 0.0891 | 0.0526102 |
| GTEx | tibial nerve | NDUFA6 | rs12160751 | 22 | 41584351 | T | C | 0.2941 | 17.19135531 | 0.145022 | 0.0349767 | 4.20E-05 | -0.0071 | 0.0307 | 0.8182 |
| GTEx | tibial nerve | NDUFA6 | rs139043947 | 22 | 42480421 | A | G | 0.02065 | 624.6358366 | 1.37241 | 0.0549124 | 2.66E-81 | 0.1497 | 0.0971 | 0.1231 |
| GTEx | tibial nerve | NDUFA6 | rs146824396 | 22 | 41922643 | C | T | 0.01194 | 69.85100957 | 1.01276 | 0.121177 | 1.33E-15 | 0.0927 | 0.129 | 0.4725 |
| GTEx | tibial nerve | NDUFA6 | rs183413369 | 22 | 41808162 | A | G | 0.0119 | 38.05495923 | 0.827739 | 0.13418 | 1.81E-09 | 0.0776 | 0.1293 | 0.5486 |
| GTEx | tibial nerve | NDUFA6 | rs6002546 | 22 | 42333408 | G | T | 0.2127 | 31.89477272 | 0.221608 | 0.0392397 | 3.26E-08 | 0.0282 | 0.0342 | 0.41 |
| GTEx | tibial nerve | NDUFA6 | rs73163373 | 22 | 42232588 | A | G | 0.1139 | 24.92728823 | 0.234662 | 0.0470008 | 9.21E-07 | 0.0272 | 0.044 | 0.5362 |
| GTEx | tibial nerve | NDUFA6 | rs73419054 | 22 | 42866999 | G | T | 0.02968 | 44.70465807 | 0.485485 | 0.0726105 | 8.52E-11 | 0.2284 | 0.0821 | 0.00538295 |
| GTEx | tibial nerve | NDUFA6 | rs742086 | 22 | 42552253 | G | T | 0.1702 | 23.0725118 | -0.1748 | 0.036391 | 2.27E-06 | -0.0209 | 0.0376 | 0.579301 |

**Supplementary Table 14. The results of colocalization analysis.**

| **Data source** | **Tissue** | **Gene** | **Outcome** | **p1** | **p2** | **p12** | **Chr** | **min.pos** | **max.pos** | **SNPs** | **SNP.PP.H4>0.75** |
| --- | --- | --- | --- | --- | --- | --- | --- | --- | --- | --- | --- |
| eQTLGen | blood | CYB5B | Aortic aneurysms | 1.00E-04 | 1.00E-04 | 1.00E-05 | 16 | 68510995 | 70349147 | 534 | rs72797202, 0.7694159 |
| eQTLGen | blood | NDUFA6 | Aortic aneurysms | 1.00E-04 | 1.00E-04 | 1.00E-05 | 22 | 41484408 | 43472319 | 1340 | rs117529804, 0.9943146 |
| GTEx | blood | CYB5B | Aortic aneurysms | 1.00E-04 | 1.00E-04 | 1.00E-05 | 16 | 69437224 | 69508496 | 73 | 0 |
| GTEx | blood | NDUFA6 | Aortic aneurysms | 1.00E-04 | 1.00E-04 | 1.00E-05 | 22 | 41588798 | 42891812 | 372 | 0 |
| GTEx | aorta | NDUFA6 | Aortic aneurysms | 1.00E-04 | 1.00E-04 | 1.00E-05 | 22 | 41541882 | 42866999 | 105 | 0 |
| GTEx | tibial artery | NDUFA6 | Aortic aneurysms | 1.00E-04 | 1.00E-04 | 1.00E-05 | 22 | 41493988 | 42871115 | 431 | rs1989202, 1 |
| GTEx | coronary artery | NDUFA6 | Aortic aneurysms | 1.00E-04 | 1.00E-04 | 1.00E-05 | 22 | 42262411 | 42866999 | 27 | 0 |
| GTEx | visceral omental adipose | NDUFA6 | Aortic aneurysms | 1.00E-04 | 1.00E-04 | 1.00E-05 | 22 | 41588798 | 42866999 | 304 | rs1989202，1 |
| GTEx | tibial nerve | NDUFA6 | Aortic aneurysms | 1.00E-04 | 1.00E-04 | 1.00E-05 | 22 | 41493988 | 42866999 | 292 | 0 |

**Supplementary Table 15. STROBE-MR checklist of recommended items to address in reports of Mendelian randomization studies**^1^ ^2^

| **Item No.** | **Section** | **Checklist item** | **Page No.** | **Relevant text from manuscript** |
| --- | --- | --- | --- | --- |
| 1 | **TITLE and ABSTRACT** | Indicate Mendelian randomization (MR) as the study’s design in the title and/or the abstract if that is a main purpose of the study | 1,2 | Two-sample MR and multivariable MR analyses were conducted to elucidate the association between metformin and AA. Colocalization analysis was employed to ascertain the probability of shared causal genetic variants between single nucleotide polymorphisms (SNPs) associated with eQTLs and AA. |
|  | **INTRODUCTION** |  |  |  |
| 2 | **Background** | Explain the scientific background and rationale for the reported study. What is the exposure? Is a potential causal relationship between exposure and outcome plausible? Justify why MR is a helpful method to address the study question | 1-2 | Introduction paragraph 2-3 |
| 3 | **Objectives** | State specific objectives clearly, including pre-specified causal hypotheses (if any). State that MR is a method that, under specific assumptions, intends to estimate causal effects | 2 | Introduction paragraph 4 |
|  | **METHODS** |  |  |  |
| 4 | **Study design and data sources** | Present key elements of the study design early in the article. Consider including a table listing sources of data for all phases of the study. For each data source contributing to the analysis, describe the following: |  |  |
|  | a) | Setting: Describe the study design and the underlying population, if possible. Describe the setting, locations, and relevant dates, including periods of recruitment, exposure, follow-up, and data collection, when available. | 2 | Paragraph “Study Design”  Fig.1 |
|  | b) | Participants: Give the eligibility criteria, and the sources and methods of selection of participants. Report the sample size, and whether any power or sample size calculations were carried out prior to the main analysis | 2-3 | Paragraph “Data sources and Selection of instrumental variables”  Supplementary Table 1 |
|  | c) | Describe measurement, quality control and selection of genetic variants | 2-3 | Paragraph “Data sources and Selection of instrumental variables” |
|  | d) | For each exposure, outcome, and other relevant variables, describe methods of assessment and diagnostic criteria for diseases | 2-3 | Paragraph “Data sources and Selection of instrumental variables” |
|  | e) | Provide details of ethics committee approval and participant informed consent, if relevant |  | This study was based on aggregate data and  therefore no institutional review board  approval was necessary. All prior studies  providing aggregate data received ethical  approval and obtained informed consent  from all participants. |
| 5 | **Assumptions** | Explicitly state the three core IV assumptions for the main analysis (relevance, independence and exclusion restriction) as well assumptions for any additional or sensitivity analysis | 3 | Paragraph “Mendelian Randomization Analysis” |
| 6 | **Statistical methods: main analysis** | Describe statistical methods and statistics used |  |  |
|  | a) | Describe how quantitative variables were handled in the analyses (i.e., scale, units, model) | 2-3 | Paragraph “Data sources and Selection of instrumental variables” |
|  | b) | Describe how genetic variants were handled in the analyses and, if applicable, how their weights were selected | 2-3 | Paragraph “Data sources and Selection of instrumental variables” |
|  | c) | Describe the MR estimator (e.g. two-stage least squares, Wald ratio) and related statistics. Detail the included covariates and, in case of two-sample MR, whether the same covariate set was used for adjustment in the two samples | 3 | Paragraph “Mendelian Randomization Analysis” |
|  | d) | Explain how missing data were addressed | / | / |
|  | e) | If applicable, indicate how multiple testing was addressed | / | / |
| 7 | **Assessment of assumptions** | Describe any methods or prior knowledge used to assess the assumptions or justify their validity | 2-3 | Paragraph “Data sources and Selection of instrumental variables” |
| 8 | **Sensitivity analyses and additional analyses** | Describe any sensitivity analyses or additional analyses performed (e.g. comparison of effect estimates from different approaches, independent replication, bias analytic techniques, validation of instruments, simulations) | 3-4 | Paragraph “Sensitivity Analysis” |
| 9 | **Software and pre-registration** |  |  |  |
|  | a) | Name statistical software and package(s), including version and settings used | 4 | Paragraph “R packages” |
|  | b) | State whether the study protocol and details were pre-registered (as well as when and where) | / | / |
|  | **RESULTS** |  |  |  |
| 10 | **Descriptive data** |  |  |  |
|  | a) | Report the numbers of individuals at each stage of included studies and reasons for exclusion. Consider use of a flow diagram | 2-3 | Paragraph “Data sources and Selection of instrumental variables”  Supplementary Table 5  Supplementary Table 13 |
|  | b) | Report summary statistics for phenotypic exposure(s), outcome(s), and other relevant variables (e.g. means, SDs, proportions) | 2-3 | Paragraph “Data sources and Selection of instrumental variables”  Supplementary Table 5  Supplementary Table 13 |
|  | c) | If the data sources include meta-analyses of previous studies, provide the assessments of heterogeneity across these studies | / | / |
|  | d) | For two-sample MR:  i.  Provide justification of the similarity of the genetic variant-exposure associations between the exposure and outcome samples  ii.  Provide information on the number of individuals who overlap between the exposure and outcome studies |  | These was no known sample overlap  between studied jointly analyzed with MR. |
| 11 | **Main results** |  |  |  |
|  | a) | Report the associations between genetic variant and exposure, and between genetic variant and outcome, preferably on an interpretable scale | 6 | Result paragraph 4  Table 2 |
|  | b) | Report MR estimates of the relationship between exposure and outcome, and the measures of uncertainty from the MR analysis, on an interpretable scale, such as odds ratio or relative risk per SD difference | 4-6 | Result paragraph 1-3  Fig.2, Fig.3,  Supplementary Table 2,4,7,9 |
|  | c) | If relevant, consider translating estimates of relative risk into absolute risk for a meaningful time period | / | / |
|  | d) | Consider plots to visualize results (e.g. forest plot, scatterplot of associations between genetic variants and outcome versus between genetic variants and exposure) |  | Supplementary Fig. 1. |
| 12 | **Assessment of assumptions** |  |  |  |
|  | a) | Report the assessment of the validity of the assumptions | 4-6 | Result paragraph 1-3  Supplementary Table 3,7,9 |
|  | b) | Report any additional statistics (e.g., assessments of heterogeneity across genetic variants, such as *I^2^*, Q statistic or E-value) |  | Supplementary Table 3,8,10 |
| 13 | **Sensitivity analyses and additional analyses** |  |  |  |
|  | a) | Report any sensitivity analyses to assess the robustness of the main results to violations of the assumptions | 4-6 | Result paragraph 1-3  Supplementary Table 3,8,10 |
|  | b) | Report results from other sensitivity analyses or additional analyses | 4-6 | Result paragraph 1-3  Supplementary Table 3,8,10 |
|  | c) | Report any assessment of direction of causal relationship (e.g., bidirectional MR) | 4-6 | Result paragraph 1-3  Supplementary Table 3,8,10 |
|  | d) | When relevant, report and compare with estimates from non-MR analyses | / | / |
|  | e) | Consider additional plots to visualize results (e.g., leave-one-out analyses) |  | Supplementary Fig. 1. |
|  | **DISCUSSION** |  |  |  |
| 14 | **Key results** | Summarize key results with reference to study objectives | 8 | Discuss paragraph 1 |
| 15 | **Limitations** | Discuss limitations of the study, taking into account the validity of the IV assumptions, other sources of potential bias, and imprecision. Discuss both direction and magnitude of any potential bias and any efforts to address them | 9-10 | Discuss paragraph 10 |
| 16 | **Interpretation** |  |  |  |
|  | a) | Meaning: Give a cautious overall interpretation of results in the context of their limitations and in comparison with other studies | 7 | Discuss paragraph 2-3 |
|  | b) | Mechanism: Discuss underlying biological mechanisms that could drive a potential causal relationship between the investigated exposure and the outcome, and whether the gene-environment equivalence assumption is reasonable. Use causal language carefully, clarifying that IV estimates may provide causal effects only under certain assumptions | 7-8 | Discuss paragraph 4-8 |
|  | c) | Clinical relevance: Discuss whether the results have clinical or public policy relevance, and to what extent they inform effect sizes of possible interventions | 8 | Discuss paragraph 11 |
| 17 | **Generalizability** | Discuss the generalizability of the study results (a) to other populations, (b) across other exposure periods/timings, and (c) across other levels of exposure | 8 | Discuss paragraph 10 |
|  | **OTHER INFORMATION** |  |  |  |
| 18 | **Funding** | Describe sources of funding and the role of funders in the present study and, if applicable, sources of funding for the databases and original study or studies on which the present study is based | Title page | Paragraph “Funding” |
| 19 | **Data and data sharing** | Provide the data used to perform all analyses or report where and how the data can be accessed, and reference these sources in the article. Provide the statistical code needed to reproduce the results in the article, or report whether the code is publicly accessible and if so, where | Title Page | Paragraph “Data Availability” |
| 20 | **Conflicts of Interest** | All authors should declare all potential conflicts of interest | Title Page | Paragraph “Competing Interests” |

This checklist is copyrighted by the Equator Network under the Creative Commons Attribution 3.0 Unported (CC BY 3.0) license.

1. Skrivankova VW, Richmond RC, Woolf BAR, Yarmolinsky J, Davies NM, Swanson SA, et al. Strengthening the Reporting of Observational Studies in Epidemiology using Mendelian Randomization (STROBE-MR) Statement. JAMA. 2021;under review.

2. Skrivankova VW, Richmond RC, Woolf BAR, Davies NM, Swanson SA, VanderWeele TJ, et al. Strengthening the Reporting of Observational Studies in Epidemiology using Mendelian Randomisation (STROBE-MR): Explanation and Elaboration. BMJ. 2021;375:n2233.


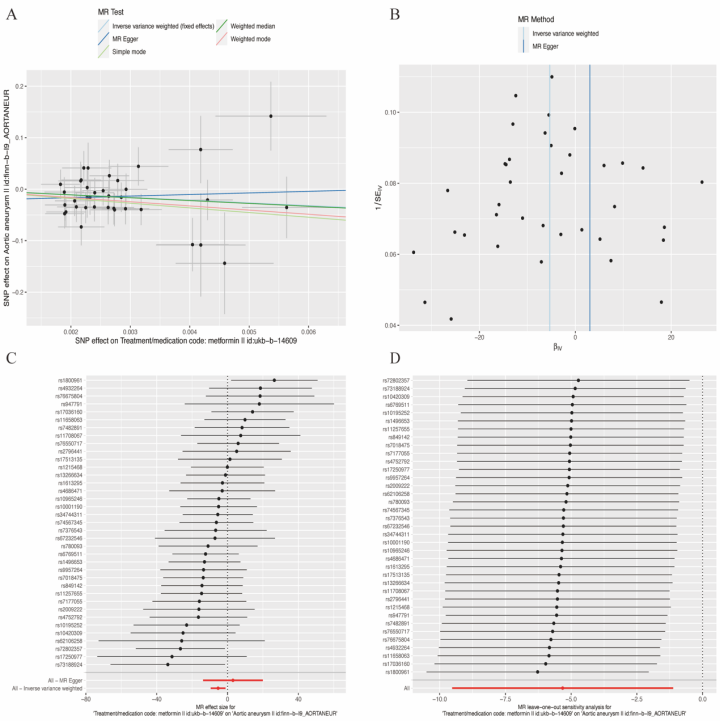


**Supplementary Fig. 1. MR effect size for causal associations of metformin use and aortic aneurysm.** (A) Scatter plot; (B) Funnel plot; (C) Forest plot; (D) Leave-one-out plot.
